# Genetically Defined Subtypes of Somatostatin-Containing Cortical Interneurons

**DOI:** 10.1523/ENEURO.0204-23.2023

**Published:** 2023-08-08

**Authors:** Rachel E. Hostetler, Hang Hu, Ariel Agmon

**Affiliations:** Department of Neuroscience, West Virginia University Rockefeller Neuroscience Institute, Morgantown, WV 26506

**Keywords:** barrel cortex, cell types, interneurons, Martinotti, somatostatin

## Abstract

Inhibitory interneurons play a crucial role in proper development and function of the mammalian cerebral cortex. Of the different inhibitory subclasses, dendritic-targeting, somatostatin-containing (SOM) interneurons may be the most diverse. Earlier studies used GFP-expressing and recombinase-expressing mouse lines to characterize genetically defined subtypes of SOM interneurons by morphologic, electrophysiological, and neurochemical properties. More recently, large-scale studies classified SOM interneurons into 13 morpho-electric transcriptomic (MET) types. It remains unclear, however, how these various classification schemes relate to each other, and experimental access to MET types has been limited by the scarcity of specific mouse driver lines. To address these issues, we crossed Flp and Cre driver lines with a dual-color intersectional reporter, allowing experimental access to several combinatorially defined SOM subsets. Brains from adult mice of both sexes were retrogradely dye labeled from the pial surface to identify layer 1-projecting neurons and immunostained against several marker proteins, revealing correlations between genetic label, axonal target, and marker protein expression in the same neurons. Lastly, using whole-cell recordings *ex vivo*, we analyzed and compared electrophysiological properties between different intersectional subsets. We identified two layer 1-targeting subtypes with nonoverlapping marker protein expression and electrophysiological properties, which, together with a previously characterized layer 4-targeting subtype, account for >50% of all layer 5 SOM cells and >40% of all SOM cells, and appear to map onto 5 of the 13 MET types. Genetic access to these subtypes will allow researchers to determine their synaptic inputs and outputs and uncover their roles in cortical computations and animal behavior.

## Visual Abstract

**Figure:**
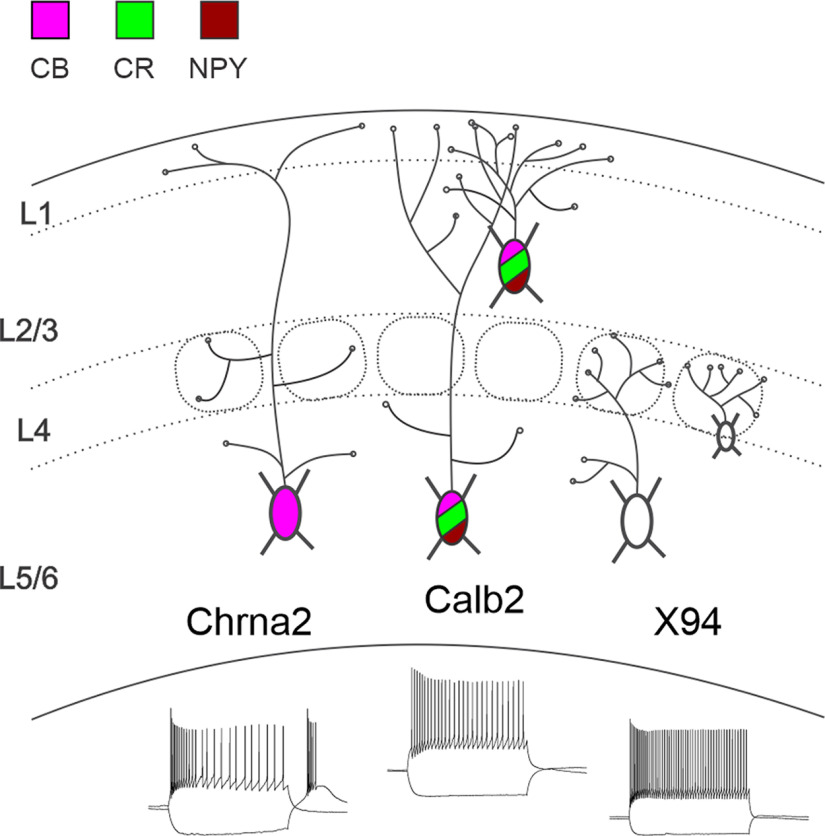

## Significance Statement

Inhibitory neurons are critically important for the proper development and function of the cerebral cortex. Although a minority population, they are highly diverse, which poses a major challenge to investigating their contributions to cortical computations and animal and human behavior. As a step toward understanding this diversity, we crossed genetically modified mouse lines to allow detailed examination of combinatorially defined groups of somatostatin-containing interneurons. We identified and characterized three somatostatin-containing subtypes in the deep cortical layers with distinct anatomic, neurochemical, and electrophysiological properties. Future studies could now use these genetic tools to examine how these different subtypes are integrated into the cortical circuit and what roles they play during sensory perception, cognitive function or motor behavior.

## Introduction

In humans, as in other mammals, the neocortex is where incoming sensory information relayed from the sensory periphery via the thalamus is processed and perceived, where decisions about appropriate motor responses are made, and where such motor actions are planned and controlled. While the majority of cortical neurons are excitatory pyramidal or spiny stellate cells, the minority inhibitory interneurons are crucial for proper neocortical development and function (Isaacson and Scanziani, 2011; Le Magueresse and Monyer, 2013; Kepecs and Fishell, 2014). Cortical interneurons fall into four main nonoverlapping subclasses characterized by the expression of the proteins parvalbumin (PV), somatostatin, or vasointestinal protein (VIP), or of the gene inhibitor of DNA binding 2 (*Id2*) (Rudy et al., 2011; Tremblay et al., 2016; Schuman et al., 2019; Machold et al., 2022). In contrast to soma-targeting, proximal dendrite-targeting, or axon initial segment-targeting PV cells, which can powerfully silence the final spike output of a neuron, SOM inhibition is more subtle, as it targets distal dendrites (Freund and Buzsáki, 1996; Kawaguchi and Kubota, 1996; Wang et al., 2004; Bloss et al., 2016), where it can affect the integration of synaptic inputs and dampen dendritic excitability (Jadi et al., 2012; Lovett-Barron et al., 2012; Doron et al., 2017). Among functions ascribed to the SOM subclass are generating surround suppression (Adesnik et al., 2012; Kato et al., 2017; Lakunina et al., 2020), promoting long-range coherence and sensory-evoked oscillations (Chen et al., 2017; Veit et al., 2017; Abbas et al., 2018; Hakim et al., 2018), and enabling learning and memory (Makino and Komiyama, 2015; Adler et al., 2019; Artinian et al., 2019; Cummings and Clem, 2020; Dobrzanski et al., 2022). Moreover, dysfunction of SOM interneurons is implicated in a variety of neuropsychiatric and neurodevelopmental disorders (Paluszkiewicz et al., 2011; Fee et al., 2017; Chen et al., 2019; Anderson et al., 2020; Zorrilla de San Martin et al., 2020; Wengert et al., 2021; He et al., 2022).

SOM interneurons may be the most diverse of the four inhibitory subclasses. Previous studies using various transgenic mouse lines identified several genetically defined SOM subsets with distinct morphologic, neurochemical, and electrophysiological phenotypes. These included GFP-expressing, layer 4 (L4)-projecting (non-Martinotti) cells in the X94 mouse line (Ma et al., 2006); GFP-expressing, layer 1-projecting (Martinotti) cells in the GIN and X98 mouse lines (Oliva et al., 2000; Ma et al., 2006); and Cre-expressing Martinotti cells in the Chrna2-Cre, Calb1-Cre, and Calb2-Cre mouse lines (He et al., 2016; Hilscher et al., 2017; Nigro et al., 2018). In recent years, the recognition of SOM diversity was reinforced by large-scale transcriptomic taxonomies. For example, a multimodal classification study (Gouwens et al., 2020) clustered SOM interneurons into 13 morpho-electric transcriptomic (MET) types but identified only 15 such types in all other inhibitory subclasses combined. Unfortunately, much of this diversity remains inaccessible to experimenters for lack of genetic targeting tools. Consequently, the great majority of the many studies to date examining the roles played by SOM interneurons in sensory processing, motor skill acquisition, and associative learning targeted the SOM subclass en masse, using the Sst-IRES-Cre line (Taniguchi et al., 2011). This line labels all SOM interneurons nonselectively and can also induce some off-target recombination (Hu et al., 2013; Mikulovic et al., 2015; Müller-Komorowska et al., 2020). Clearly, there is a major gap between our recognition of the transcriptomic and phenotypic diversity of SOM interneurons and our ability to selectively target specific subtypes for recording, imaging, or activity manipulations.

To begin to close this gap, we characterized in detail SOM subsets captured by combinatorial breeding of five driver and two transgenic mouse lines. In adult mice of both sexes, we identified three nonoverlapping SOM subtypes with distinct axonal targets, marker protein expression, and electrophysiological properties, which, together, account for over half of all SOM interneurons in L5 and for >40% of all SOM interneurons. Our findings call for a renewed effort to generate additional driver lines that can be used combinatorially to provide experimental access to additional SOM subtypes, to characterize the intrinsic properties and synaptic connectivity patterns of these subtypes, and to uncover their roles in cortical computations and behavior.

## Materials and Methods

### Animal welfare

Animals used in this study were housed at the Association for Assessment and Accreditation of Laboratory Animal Care International (AAALAC)-accredited West Virginia University (WVU) Lab Animal Research Facility according to institutional, federal, and AAALAC guidelines. Animals were housed in group cages unless breeding, pregnant, nursing, or after a surgical procedure, and were provided with environmental enrichment items. Brain slice preparation and perfusion fixation were conducted under deep anesthesia. All animal procedures followed the Public Health Service Policy on Humane Care and Use of Laboratory Animals and the Society for Neuroscience Policy on the Use of Animals in Research, and were approved by the WVU Institutional Animal Care and Use Committee.

### Mouse strains

To label genetically distinct subsets of SOM interneurons, we crossed Sst-Flp mice (catalog #028579, The Jackson Laboratory; He et al., 2016) with one of the following four Cre recombinase-expressing mouse lines: Calb2-Cre (strain #010774, The Jackson Laboratory; Taniguchi et al., 2011), Calb1-Cre (strain #028532, The Jackson Laboratory; Daigle et al., 2018), Chrna2-Cre (Leão et al., 2012), and Pdyn-Cre (strain #027958, The Jackson Laboratory; Krashes et al., 2014). Dual-recombinase progeny were then crossed with the RC::FLTG reporter line (strain #026932, The Jackson Laboratory; Plummer et al., 2015) to create triple-transgenic mice expressing GFP in Cre^+^/Flp^+^ cells and tdTomato in Cre^–^/Flp^+^ cells. We also used X94 and X98 mice (strains #006340 and #006334, The Jackson Laboratory; Ma et al., 2006) to label previously characterized subsets of L4-projecting and L1-projecting SOM cells, respectively, crossing them with Cre driver lines and the Ai9 tdTomato reporter (strain #007909, The Jackson Laboratory; Madisen et al., 2010).

### Retrograde labeling

Mice used for histologic experiments were 1–5 months old (typically, 2–3 months old), of both sexes. Mice were deeply anesthetized with isoflurane at zeitgeber time 6 (ZT6) to ZT10, placed in a heated stereotactic frame, and injected subcutaneously with local anesthetic (bupivacaine) and analgesic (meloxicam). The skull over the right primary somatosensory (S1) cortex (barrel cortex) was exposed, and a flap of bone (∼2 × 3 mm) was outlined with a 0.25 mm drill and removed together with the dura mater. A filter paper circle, presaturated with Fast Blue dye solution (FB; Polysciences; 1% in distilled water) and allowed to dry, was cut to size, dipped in cortex buffer (composition: 125 mm NaCl, 5 mm KCl, 10 mm glucose, 10 mm HEPES, 2 mm CaCl_2_, and 2 mm MgSO_4_), placed over the exposed pial surface, and covered with Kwik-Cast silicon sealant (World Precision Instruments). The skin incision was then closed, and mice were allowed to recover. Criteria for successful retrograde labeling are indicated below.

### Histology

One day (24 ± 2 h) after surgery, mice were deeply anesthetized with an intraperitoneal injection of Avertin and transcardially perfused with ∼30 ml of room temperature saline followed by 50 ml of room temperature 4% paraformaldehyde (PFA) at 5 ml/min. Brains were removed and postfixed in 4% PFA at room temperature for 4 h on a shaker plate, then placed in 30% sucrose in PBS at 4°C on a shaker plate for at least 1–2 d. Brains were sectioned through the barrel cortex on a freezing microtome (−30°C) in the coronal plane at a thickness of 30 μm (60 μm for X94 mice). Every FB-labeled tissue section within barrel cortex was collected. For immunostaining, every fourth section was immunostained with each antibody. In total, 4–12 sections were collected per brain, and typically 4 sections were stained with each antibody.

### Immunocytochemistry

Free-floating fixed sections were blocked in 5% goat serum, 0.5% Triton X-100 (TX) in PBS for 1 h at room temperature, and then were incubated with primary antibody in 1.25% goat serum and 0.125% TX in PBS for 48 h at 4°C. Sections were then washed 3× with PBS and incubated with secondary antibodies in 1% goat serum and 0.1% TX in PBS for 2 h at room temperature. Finally, sections were washed 3× with PBS and mounted in Vectashield Antifade (Vector Laboratories) or Prolong Diamond (Thermo Fisher Scientific) mounting medium. Sections stained with mouse primary antibodies were blocked in ReadyProbes “Mouse on Mouse” IgG blocking solution (Thermo Fisher Scientific) for 1 h before the initial blocking step. Primary antibodies and dilutions used were rabbit anti-calretinin (CR; 1:2000; Swant), mouse anti-calbindin (CB; 1:500; Swant), and rabbit anti-neuropeptide Y (NPY; 1:2000; Immunostar). Secondary antibodies used were Alexa Fluor 647 anti-rabbit (1:1000; Thermo Fisher Scientific) and Alexa Fluor 647 anti-mouse (1:500; Thermo Fisher Scientific).

### Confocal imaging and histologic analysis

The same sections were used to analyze fluorescent protein expression, FB labeling, and immunostaining. Images of stained sections were taken on an inverted confocal microscope (model A1R, Nikon) using a 20×, numerical aperture (NA) 0.75 objective, at a *z*-step of 2.5 μm. Lasers of 405, 488, 561, and 640 nm wavelengths were used to excite FB, GFP, TdTomato and Alexa Fluor 647, respectively. In each section, a region of interest (ROI) encompassing the cortical region underlying the FB deposit, typically 700–1000 μ;m wide, was selected, and all SOM interneurons within it (identified by GFP or tdTomato expression) were digitally marked, using NIS Elements (Nikon), as positive or negative for FB and for the relevant antibody, by visually inspecting the full confocal *z*-stack. Cortical layers were determined by cell body shape, size, and density. Only brains with successful retrograde labeling were included in this and subsequent analysis; criteria for successful labeling included strong FB labeling in subplate neurons (as evidence for sufficient incubation time for dye to reach all cortical layers) and largely label-free L4 (as evidence for dye uptake limited to L1). Insufficient labeling typically resulted from incomplete removal of the dura mater or from dislodging of the filter paper during the incubation period, whereas large numbers of FB-labeled cells in L4 typically reflected damage to the pial surface and diffusion of dye to L2/3. Similar selection parameters were used in previous studies using the same retrograde labeling method (Ramos-Moreno and Clascá, 2014).

### *Ex vivo* brain slice preparation

Mice of either sex, typically 1–2 months old, were decapitated at ZT4–ZT6 under deep isoflurane anesthesia, and the brains were removed and submerged in ice-cold, sucrose-based artificial CSF (ACSF) containing the following (in mm): sucrose 206, NaH_2_PO_4_ 1.25, MgCl_2_.6H_2_O 10, CaCl_2_ 0.25, KCl 2.5, NaHCO_3_ 26, and d-glucose 11, pH 7.4. Thalamocortical brain slices (Agmon and Connors, 1991; Porter et al., 2001) of somatosensory (barrel) cortex, 300–350 μm thick, were cut in the same solution using a vibratome (model VT-200, Leica), and placed in a submersion holding chamber filled with recirculated and oxygenated ACSF (in mm: NaCl 126, KCl 3, NaH_2_PO_4_ 1.25, CaCl_2_ 2, MgSO_4_ 1.3, NaHCO_3_ 26, and d-glucose 20). Slices were incubated for at least 30 min at 32°C and then at room temperature until use. For recording, individual slices were transferred to a submersion recording chamber and continuously superfused with 32°C oxygenated ACSF at a rate of 2–3 ml/min.

### Electrophysiological recordings

Recordings were performed on an upright microscope (model FN-1, Nikon) under a 40× water-immersion objective. For whole-cell recordings, glass micropipettes (typical resistance, 5–8 MΩ) were filled with an intracellular solution containing the following (in mm): K-gluconate 134, KCl 3.5, CaCl_2_ 0.1, HEPES 10, EGTA 1.1, Mg-ATP 4, phosphocreatine-Tris 10, and 2 mg/ml biocytin, adjusted to pH 7.25 and 290 mOsm. Labeled neurons were identified visually and with a digital camera (Nikon) by their GFP or tdTomato fluorescence and were targeted for single or dual whole-cell recordings using a MultiClamp 700B amplifier (Molecular Devices). Upon break-in, cells were routinely tested by a standardized family of incrementing 600-ms-long intracellular current steps in both negative and positive directions relative to resting potential. In *post hoc* analysis, the same records were used to extract multiple electrophysiological parameters for each cell (see below). Data were acquired at a 20 kHz sampling rate using a National Instruments analog-to-digital board controlled by an in-house acquisition software written in the LabView (National Instruments) environment. Reported intracellular voltages are not corrected for liquid junction potential.

### Electrophysiological parameters definitions

A total of 10 electrophysiological parameters were measured or calculated per cell. Rheobase was determined from a series of current steps increasing in 8–12 pA increments. *I_max_* was the maximal current step applied just below the level inducing depolarization block, evident as spike dropouts or noticeably reduced spike amplitudes.

Parameter definitions are as follows: *V*_rest_, resting potential immediately after break-in, with no holding current applied; *V*_threshold_, the transmembrane voltage when dv/dt (the slope of membrane depolarization) reached 5 V/s, measured at rheobase; *spike height*, spike peak – *V*_threshold_, measured at rheobase; *SWHH* (spike width at half-height), spike width measured at rheobase, half-way between *V*_threshold_ and spike peak; *AHP*, *V*_threshold_ – spike trough, measured at rheobase; *R*_in_ (input resistance), the slope of the *I–V* plot, calculated from four to six positive and negative subthreshold current steps, at membrane potentials up to ±15 mV from rest; *sag*, the slope of the plot of voltage sag (maximum voltage – steady-state voltage) versus membrane potential, calculated from negative current steps; *F*_init_ (initial firing frequency), the firing frequency computed as the reciprocal of the average of the first three interspike intervals (ISIs) in a spike train elicited by *I*_max_, *F*_ss_ (steady-state firing frequency), the firing frequency computed as the reciprocal of the average of the last five ISIs in a spike train elicited by *I*_max_; and *AR* (adaptation ratio), *F*_ss_/*F*_init_.

### Statistical analysis

Unless noted otherwise, exact *p*-values were computed using distribution-free, nonparametric permutation tests, by performing 10,000 random permutations of the data and calculating the fraction of permutations resulting in equal or more extreme values of the relevant statistic (under both tails, except for the *F*-statistic which is one sided; Good, 1999). When no more extreme values were found, this is indicated as *p* < 0.0001. Principal component and discriminant function analyses were computed using custom routines following Manly (2005), as described in detail by Ma et al. (2006). All data are reported as the mean ± SEM, unless indicated otherwise.

### Software accessibility

All computations were programmed in MathCad; routines are available on request.

### Ethics statement

Animal husbandry and experimental procedures followed the Public Health Service Policy on Humane Care and Use of Laboratory Animals and the Society for Neuroscience Policy on the Use of Animals in Research, and were approved by the Institutional Animal Care and Use Committee. West Virginia University has Public Health Service-approved Animal Welfare Assurance D16-00362 (A3597-01).

## Results

### Targeting SOM subsets by intersectional genetics

**Figure 1.**
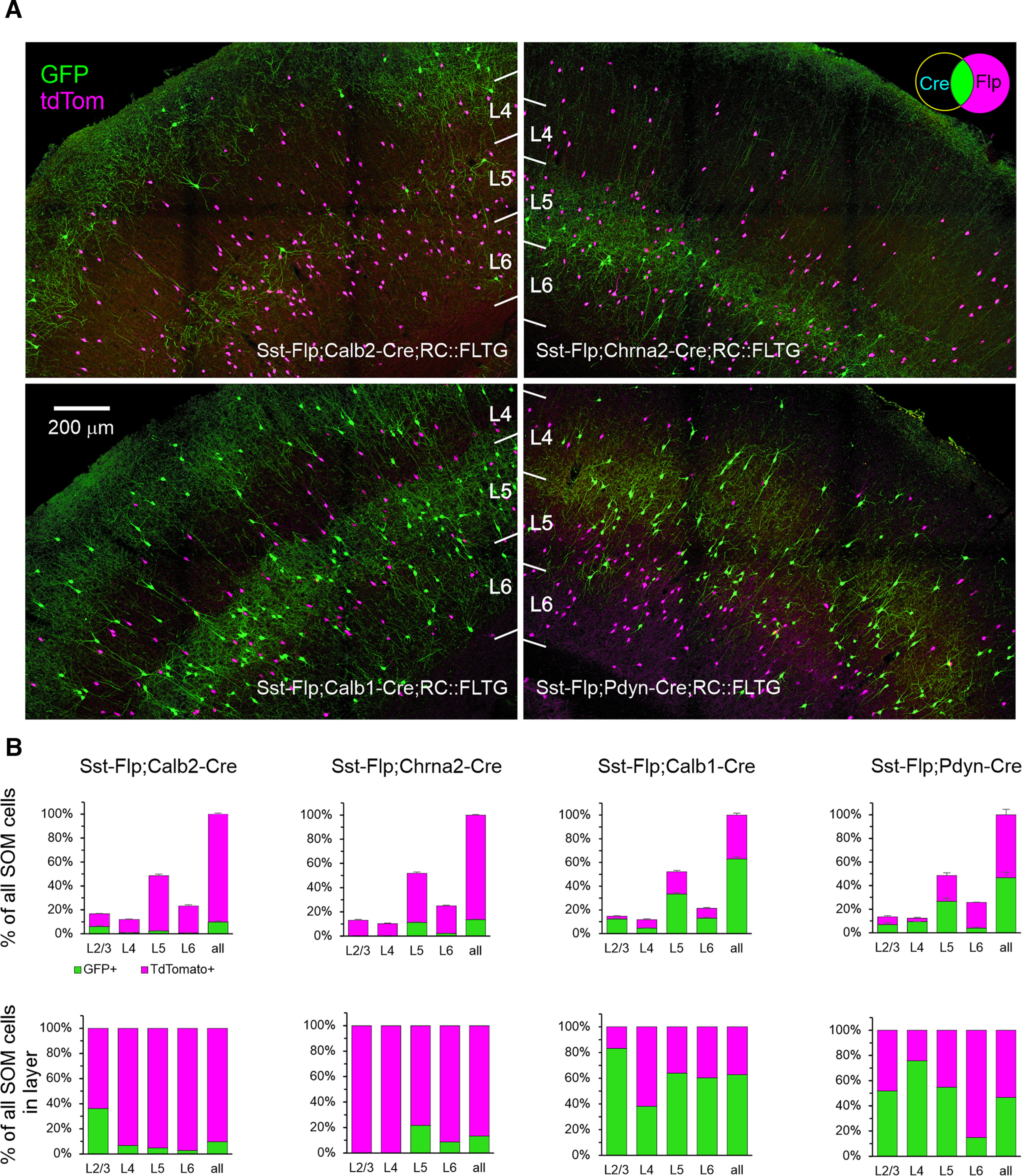
Fluorescent reporter expression in the 4 intersectional genotypes. ***A***, Representative projections of confocal *z*-stacks taken with a 20×, 0.75 NA objective at 2.5 μ;m *z*-steps through 30-μ;m-thick sections. Color channels were adjusted individually in each panel. The Venn diagram in top right illustrates the combinatorial logic of the reporter. ***B***, Top panels, TdTomato^+^ and GFP^+^ fraction of all SOM cells counted in each brain, averaged by genotype. Error bars are the SEM. Bottom panels, The same data normalized by layer. *N* = 4, 6, 4, and 3 mice for Calb2, Chrna2, Calb1, and Pdyn intersections, respectively. Numerical data are provided in Table 1.

To develop genetic tools for accessing distinct subtypes of SOM interneurons, we searched for available mouse driver lines in which Cre recombinase was coexpressed with marker genes for identified transcriptomic SOM groups (Tasic et al., 2018). We selected the following four such lines: Calb2-IRES-Cre (Taniguchi et al., 2011), shown to label CR-containing SOM cells (Taniguchi et al., 2011; He et al., 2016; Nigro et al., 2018); Chrna2-Cre (Leão et al., 2012), shown to label hippocampal oriens-lacunosum moleculare interneurons and also a subset of L5 SOM cells expressing the α2 nicotinic receptor subunit (Hilscher et al., 2017); Calb1-IRES2-Cre (Daigle et al., 2018), in which Cre is coexpressed with CB, a marker for SOM subsets (Kawaguchi and Kubota, 1997; Ma et al., 2006); and Pdyn-IRES-Cre (Krashes et al., 2014), coexpressing Cre with prodynorphin. We selected the latter line since antibodies to preprodynorphin label a subset of middle-layer SOM cells (Sohn et al., 2014), and we were looking for a line that will target the X94 (non-Martinotti) SOM subtype in these layers (Ma et al., 2006). Marker protein (and thereby Cre) expression in these lines may not be restricted to SOM interneurons; for example, CR is also expressed by VIP-containing interneurons (Gonchar et al., 2007; Xu et al., 2010), and CB is also weakly expressed by upper layer excitatory cells (van Brederode et al., 1991). Moreover, these proteins may be expressed even more widely during development (Su et al., 2021), inducing permanent recombination in non-SOM populations when crossing driver with reporter lines. We therefore resorted to an intersectional strategy (He et al., 2016; Kim et al., 2017; Nigro et al., 2018; Wu et al., 2022), but chose the following novel approach: we crossed each of the four Cre line with the Sst-Flp line and the combinatorial RC::FLTG reporter (Plummer et al., 2015), resulting in triple-transgenic progeny in which the Cre-expressing subset of SOM cells expressed GFP, while all other SOM cells (but no other cells) expressed tdTomato (Fig. 1*A*, top right, Venn diagram). For convenience, we will refer to the SOM neurons expressing GFP in the four intersectional genotypes above as Calb2, Chrna2, Calb1, and Pdyn neurons, respectively.

**Table 1:** Laminar distributions of GFP-expressing cells in the 4 intersectional subsets

		L2/3	L4	L5	L6	All layers
Calb2 (*N* = 4)	% of all SOM	6.0 (0.8)	0.8 (0.3)	2.3 (0.2)	0.6 (0.2)	9.7 (0.8)
	% in layer	36.1	6.6	4.8	2.6	9.7
Chrna2 (*N* = 6)	% of all SOM	0.0 (0.0)	0.0 (0.0)	11.2 (0.5)	2.2 (0.2)	13.4 (0.5)
	% in layer	0.2	0.0	21.7	8.8	13.4
Calb1 (*N* = 4)	% of all SOM	12.2 (0.5)	4.5 (0.3)	33.3 (1.2)	12.9 (0.9)	63.0 (1.6)
	% in layer	83.2	38.3	63.9	60.3	63.0
Pdyn (*N* = 3)	% of all SOM	7.0 (1.3)	9.3 (0.1)	26.6 (2.8)	3.8 (0.7)	46.7 (4.6)
	% in layer	51.8	75.7	54.7	14.9	46.7
All SOM (*N* = 17)	% of all SOM	14.4 (0.4)	11.4 (0.3)	50.5 (0.7)	23.8 (0.6)	100.0

For each genotype, the top row indicates the number of GFP^+^ cells in each layer as a percentage of all SOM cells (GFP^+^ or tdTomato^+^) counted in each brain, averaged over all brains of that genotype. The SEM indicated in parenthesis. The bottom row expresses the same counts as a percentage of all SOM cells in each layer. These data are plotted in Figure 1*B*. The last row of the table quantifies the distribution of all SOM cells by layer, averaged over all 17 brains.

We characterized the four intersectional genotypes by imaging and analyzing brains from 17 mice of both sexes, 1–4 months old (except for one animal >5 months old), 3–6 mice per genotype. Perfusion-fixed brains were cut into 30-μ;m-thick sections through the barrel cortex, and 4–12 sections per brain (33–36 sections/genotype, 138 sections in total) were imaged with a 20×, 0.75 NA objective on a confocal microscope, with optical sections taken at 2.5 μ;m *z*-steps. In each section, the analyzed ROI was selected based on the extent of FB labeling (see below), and typically extended 700–1000 μ;m along the pial surface, spanning pia to white matter. The same ROIs (imaged with different laser lines) were used for quantifying fluorescent protein expression, FB labeling, and immunostaining, as described below.

The pattern of fluorescent protein expression is illustrated in Figure 1*A* by a representative confocal projection from each genotype. As evident from these images, the four genotypes had very different laminar distributions of GFP-expressing cells, with the Calb2 and Calb1 cell bodies distributed in L5 and L2/3, the Pdyn subset mostly in L4 and L5 and cell bodies of Chrna2 cells restricted to a narrow band in lower L5/upper L6. Also evident is a superficial band of axonal arborizations, restricted to L1 in the Chrna2 subset but encompassing also L2/3 in the Calb2 and Calb1 subsets, in addition to dense arborizations surrounding the cell bodies in L5. In the Pdyn genotype, axonal arborizations were most prominent in L4, in sharp contrast to the other three genotypes whose axons appeared to avoid L4.

The dual-color fluorescence of the combinatorial reporter allowed us to quantify not only the laminar distribution of neurons belonging to each subset, but also their prevalence within the overall SOM population. To do so, we counted the number of GFP-expressing and tdTomato-expressing cells by layer, by visual inspection of all optical planes imaged in each section. To correct for differences in the total cortical volume analyzed in different animals (e.g., because of variations in the extent of FB labeling), cell counts in each brain were normalized to all SOM cells (both GFP expressing and tdTomato expressing) counted in that brain, and then averaged within each genotype (Fig. 1*B*, top panels, Table 1). For clarity, the same data are also shown normalized to all SOM cells within each layer (Fig. 1*B*, bottom panels). When grand averaged over all four genotypes (Table 1, last row), ∼15% of all SOM cells were found in L2/3, 10% in L4, 50% in L5, and 25% in L6. Calb2 cells comprised 10% of all SOM cells; they were found mostly in L2/3, where they represented nearly 40% of all SOM cells, with a minor population in L5 and very small numbers in L4 and L6. The Chrna2 group comprised 13% of all SOM cells; it straddled the L5/6 boundary, comprising ∼20% of L5 and ∼10% of L6 SOM cells. Calb1 cells were 63% of all SOM interneurons, ranging from 40–80% of SOM cells in different layers, with the lowest percentage in L4. Given that this subset includes 80% of all SOM cells in L2/3, it must contain at least half of all Calb2 cells in that layer. Lastly, Pdyn cells were slightly less than half of all SOM cells, comprising 75% of SOM cells in L4 and about half of SOM cells in L2/3 and L5, with a small number in L6. Our counts of the Calb1, Calb2, and Chrna2 subsets are in excellent agreement with other studies of these genetic subsets in which SOM interneurons were labeled by immunocytochemistry (Nigro et al., 2018) or by fluorescent *in situ* hybridization (Wu et al., 2022).

### Overlap of intersectional subsets with transgenic subtypes

**Figure 2.**
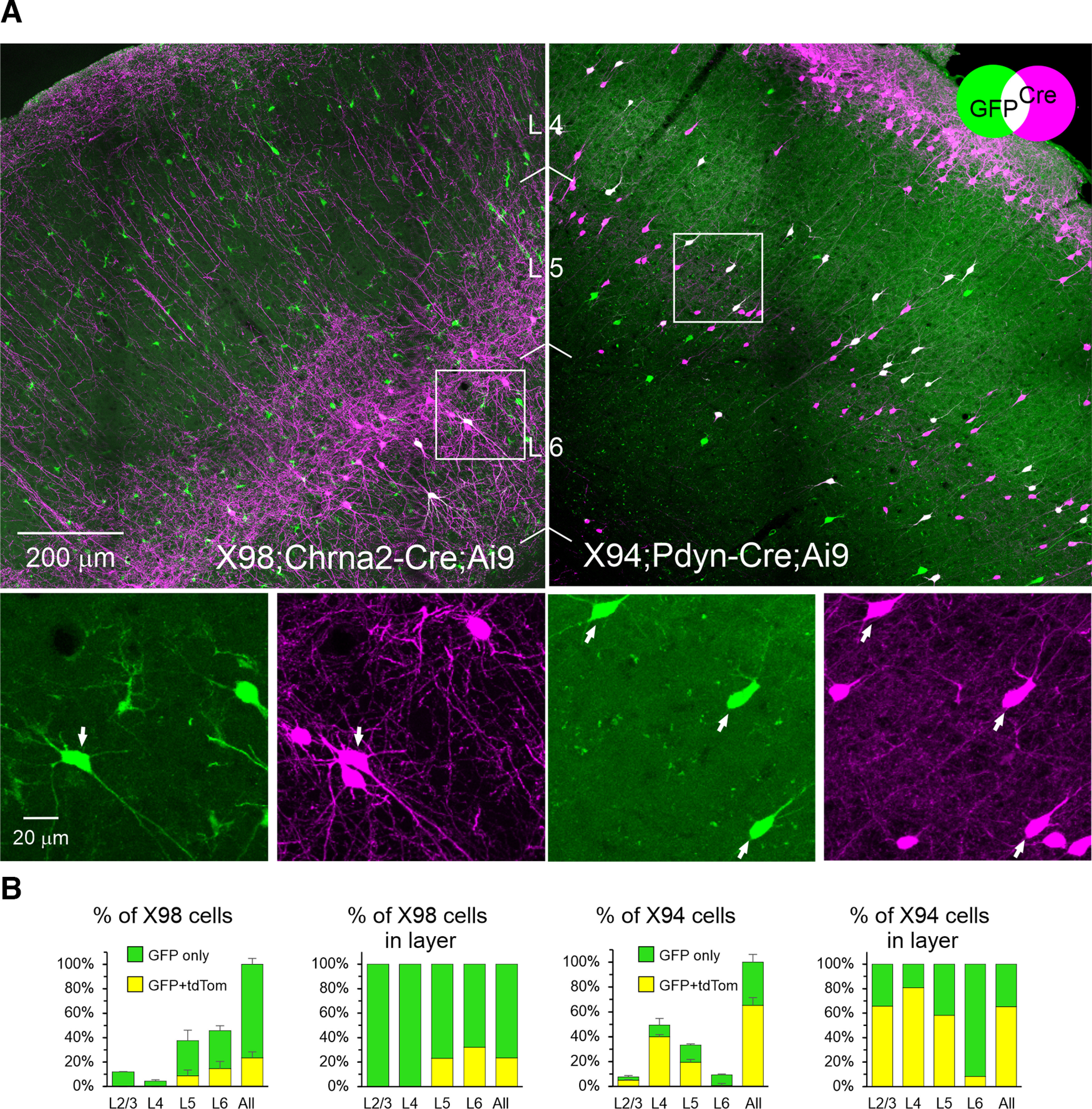
Overlap of intersectional and transgenic subsets. X98 mice were crossed with Chrna2-Cre and a tdTomato reporter, and X94 mice were crossed with Pdyn-Cre and a tdTomato reporter. ***A***, Projections of confocal *z*-stacks through representative 30-μ;m-thick sections of the indicated genotype, taken with a 20×, 0.75 NA objective at 2.5 μ;m *z*-steps. The Venn diagram illustrates the combinatorial color code. Color channels were adjusted individually in each panel. Boxed ROIs in top panels are shown at higher magnification in bottom panels, separated into the two color channels. Arrows point to double-labeled cells. In the top left panel, note a large number of non-SOM cells, many with glial morphology, weakly expressing GFP. In the top right panel, note that a considerable population of L2 pyramidal neurons also expressed Pdyn-Cre, underscoring the need for the intersectional approach used here. ***B***, Left panel of each genotype shows the fraction of GFP^+^-only and double-labeled cells (GFP^+^/tdTomato^+^) in each layer, of all GFP^+^ cells counted in each brain, averaged by genotype. Error bars are the SEM. The right panel of each genotype block shows the same counts expressed as a fraction of all GFP^+^ cells in each layer. *N* = 3, 2, for X98 and X94, respectively. Numerical data are provided in Table 2.

We previously developed and characterized two transgenic mouse lines, X98 and X94, with GFP expression restricted to specific subsets of SOM interneurons. X98 cells are L1-targeting (Martinotti) SOM neurons that reside mostly in the infragranular layers and could therefore overlap with Chrna2 neurons. X94 cells are L4-targeting (non-Martinotti) SOM neurons that reside in layers 4/5 and could therefore overlap with Pdyn neurons. To clarify the relationships between these subsets, we bred the hybrid genotypes Chrna2-Cre;X98 and Pdyn-Cre;X94, and crossed them with a Cre-dependent tdTomato reporter (Fig. 2*A*). We imaged fixed brain sections as described above and counted the fraction of X98 and X94 cells that also expressed tdTomato (Fig. 2*B*, Table 2). Less than one-quarter of all X98 cells expressed tdTomato, indicating that the X98 and Chrna2 subsets are largely nonoverlapping populations. In contrast, two-thirds of X94 cells expressed tdTomato and were thereby contained within the Pdyn subset.

**Table 2:** Overlap between intersectional and transgenic SOM subsets

		L2/3	L4	L5	L6	All layers
X98;Chrna2-Cre (*N* = 3)	% of all GFP^+^	12.1 (0.2)	4.5 (1.1)	37.7 (8.9)	45.7 (8.7)	100
	% DL of all GFP^+^	0.0	0.0	8.7 (4.8)	14.7 (5.8)	23.4 (4.9)
	% DL in layer	0.0	0.0	23.2	32.1	23.4
X94;Pdyn-Cre (*N* = 2)	% of all GFP^+^	7.8 (0.7)	49.5 (3.6)	33.4 (3.4)	9.3 (0.9)	100
	% DL of all GFP^+^	5.1 (1.0)	40.0 (5.3)	19.4 (1.0)	0.8 (0.8)	65.4 (6.2)
	% DL in layer	65.6	80.9	58.2	8.2	65.4

Counts are from the same genotypes illustrated in Figure 2. In each genotype, the top row is the distribution of GFP^+^ cells (i.e., X98 or X94) in each layer, as a percentage of all GFP^+^ cells counted in each brain, averaged by genotype. The SEM is indicated in parenthesis. Middle row, Double-labeled (DL; GFP^+^ and tdTomato^+^) cells in each layer, as a percentage of all GFP^+^ cells counted in each brain, averaged by genotype. Bottom row, The same numbers as percentage of all GFP^+^ cells in each layer. These data are plotted in Figure 2*B*.

### Classifying SOM subtypes by their L1 projection

**Figure 3.**
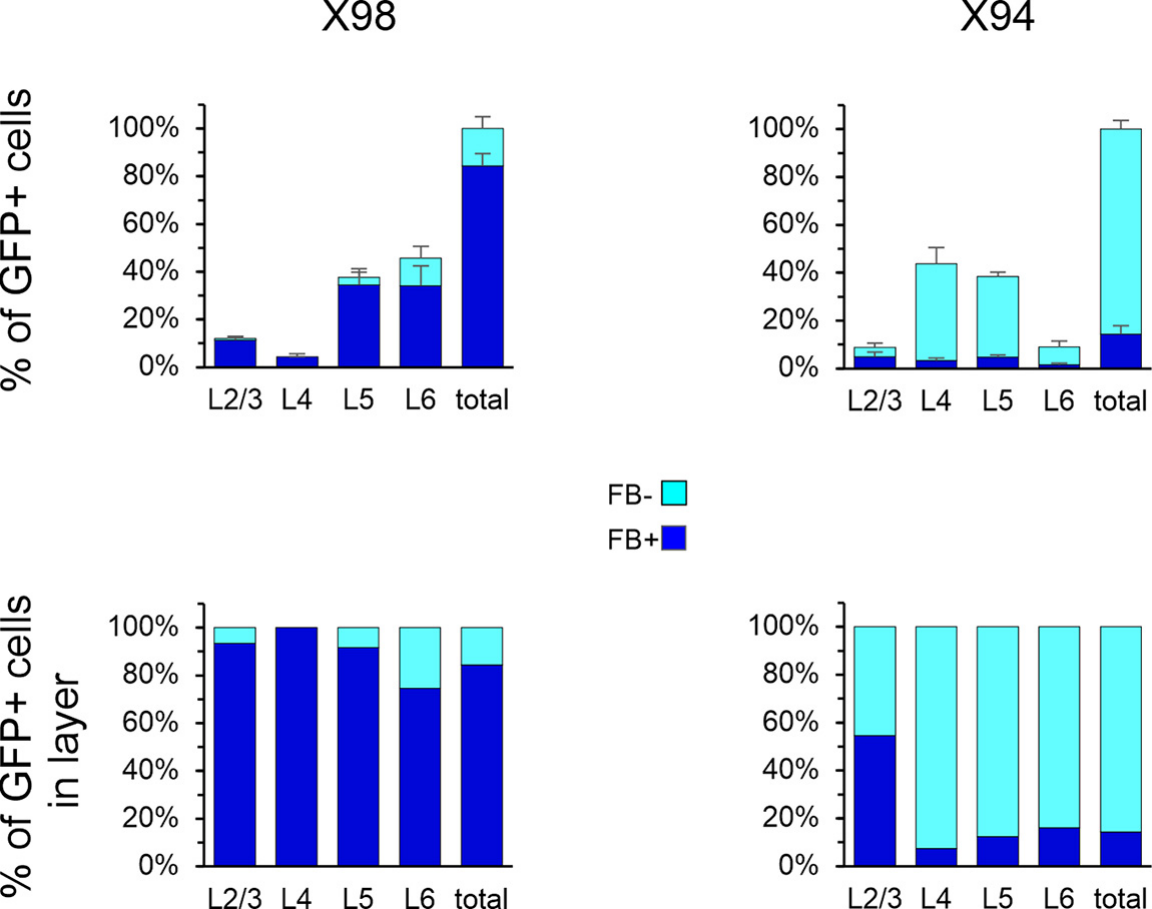
Retrograde FB labeling in the X98 and X94 subtypes. Top panels, Number of FB^+^ and FB^–^ GFP^+^ cells, as a fraction of all GFP^+^ cells counted in each brain, averaged by genotype. Bottom panels, Same numbers as a fraction of all GFP^+^ cells in each layer. Numerical data are provided in Table 3.

SOM interneurons with radially ascending axons projecting to L1 are historically referred to as Martinotti cells (Marín-Padilla, 1990; DeFelipe, 2002). Not all SOM interneurons, however, are L1 projecting; several important groups do not terminate in L1 and are collectively referred to as non-Martinotti cells (Tremblay et al., 2016). There is no quantitative estimate to date on the proportion of anatomically verified Martinotti versus non-Martinotti SOM cells in the mouse cortex. To distinguish between Martinotti and non-Martinotti cells, we retrogradely labeled SOM neurons by placing an FB-infused filter paper on the pial surface, 24 h before fixing the brains by transcardial perfusion (Cauller et al., 1998; Ramos-Moreno and Clascá, 2014). FB-labeled cells were then counted by visual inspection of 9–12 optical planes imaged through each tissue section. To validate this approach as a reliable method for labeling L1-projecting cells, but not non-L1-projecting cells, we performed retrograde labeling in mice of the X98 and X94 lines, in which GFP-expressing SOM cells are L1- and L4- projecting, respectively (Ma et al., 2006). Overall, 84 ± 5% (*N* = 3) of all X98 cells, but only 14 ± 3% (*N* = 3) of all X94 cells (7% in L4), were FB labeled (Fig. 3, Table 3), consistent with the distinct axonal projection targets of these transgenically defined SOM subsets.

**Table 3:** Retrograde FB labeling in X98 and X94 mice

		L2/3	L4	L5	L6	All layers
X98 (*N* = 3)	% FB^+^ of all GFP^+^	11.3 (0.9)	4.5 (1.1)	34.5 (6.8)	34.1 (8.4)	84.4 (5.1)
	% FB^+^ in layer	93.4	100.0	91.6	74.6	84.4
X94 (*N* = 3)	% FB^+^ of all GFP^+^	4.8 (2.0)	3.2 (1.2)	4.8 (0.9)	1.4 (0.8)	14.3 (3.5)
	% FB^+^ in layer	54.7	7.4	12.4	16.1	14.3

In each genotype, the top row is the number of FB-labeled GFP^+^ cells per layer as a percentage of all GFP^+^ cells counted in each brain, averaged by genotype. The SEM is indicated in parenthesis. Bottom row, Same numbers expressed as a percentage of all GFP^+^ cells in each layer. These data are plotted in Figure 3.

**Figure 4.**
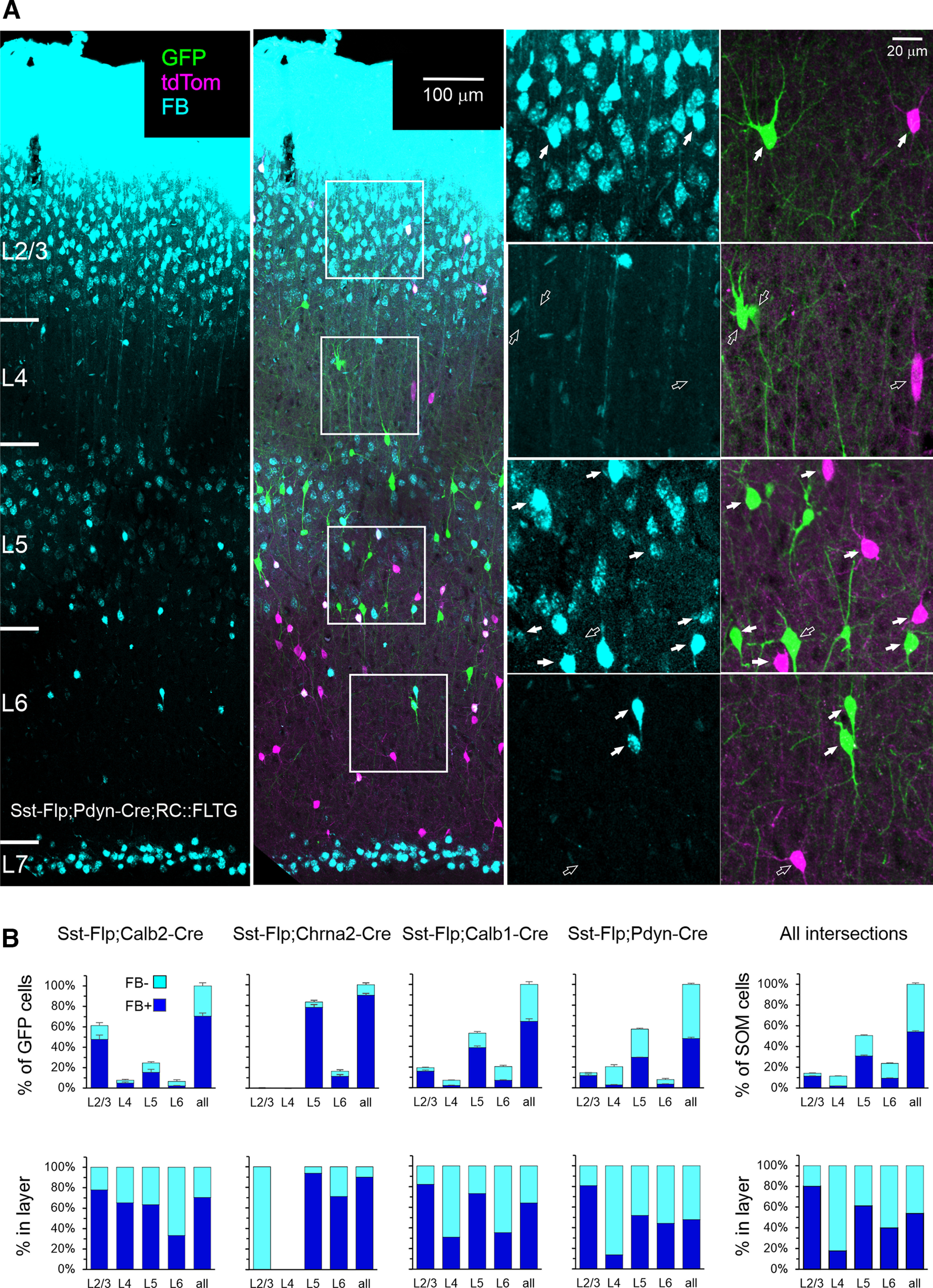
Retrograde FB labeling. ***A***, Representative 30-μ;m-thick section from a retrogradely labeled Pdyn mouse. Image is a projection of a *z*-stack taken with a 20×, 0.75 NA objective at 2.5 μ;m *z*-steps; color channels were adjusted individually in each panel. Far left panel shows only the FB color channel, and the adjacent panel also shows GFP and tdTomato channels. The 4 boxed ROIs are shown enlarged to the right, separated into the FB channel (left panels) and GFP-tdTomato channels (right panels). Filled arrows point to all FB^+^ SOM cells in each ROI, hollow arrows point to all FB^–^ SOM cells. ***B***, Top panels, Number of FB^+^ and FB^–^ GFP^+^ cells as a percentage of all GFP^+^ cells counted in each brain, averaged by genotype. Bottom panels, Same numbers as a percentage of all GFP^+^ cells in each layer. Error bars are the SEM. Plots at the far right show the fraction of FB^+^ cells of all SOM cells in all genotypes. The number of animals is as in Figure 1. Numerical data are provided in Table 4.

A confocal projection through a representative section from a retrogradely labeled Sst-Flp;Pdyn-Cre;RC::FLTG brain is illustrated in Figure 4*A*, with the full cortical depth shown in the left panels and four selected ROIs (from L2/3 to L6) magnified in the right panels. As seen in these images, the majority of FB-labeled cells in L2/3 and in L5 exhibited pyramidal morphology and were most likely pyramidal cells labeled via their axonal terminations or dendritic tufts in L1. In contrast, L4 and L6 were mostly devoid of label, as excitatory neurons in these layers rarely extend dendrites or axons to L1 (Thomson, 2010; Oberlaender et al., 2012; Wang et al., 2018). Notably, a thin layer of cells abutting the subcortical white matter, in L6B (also referred to as L7; Reep, 2000), were found to be brightly labeled, as previously observed after pial dye deposits (Mitchell and Cauller, 2001; Ramos-Moreno and Clascá, 2014). This robust label in the deepest cortical layer indicated that the 24 h survival time in our experiments was sufficient to retrogradely label any cortical neuron with an axonal projection in L1. In each section we characterized each GFP-expressing or tdTomato-expressing cell as either FB^+^ or FB^–^. The fraction of FB^+^ cells in each genetic subset is quantified by layer in Figure 4*B*, both as a fraction of all cells of this subset (Fig. 4*B*, top panels) and normalized by layer (Fig. 4*B*, lower panels); numerical data are provided in Table 4. The majority of Calb2, Chrna2, and Calb1 cells were FB^+^ (70%, 90%, and 64%, respectively), while only about half of the Pdyn subsets were. In all subsets, nearly all FB^+^ cells were found in L2/3 and/or L5. Of all SOM cells in all layers, 54 ± 1% (*N* = 17) were retrogradely labeled and therefore were *bona fide* Martinotti cells. This fraction varied by layer, from 80% in L2/3 to ∼60% in L5, 40% in L6, and <20% in L4.

**Table 4:** Percentage of retrogradely labeled cells in the 4 intersectional subsets

		L2/3	L4	L5	L6	All layers
Calb2 (*N* = 4)	% of all GFP^+^	47.7 (4.2)	5.0 (1.1)	15.5 (2.9)	2.2 (0.5)	70.5 (3.1)
	% in layer	78.0	65.5	63.4	33.2	70.5
Chrna2 (*N* = 6)	% of all GFP^+^	0.0 (0.0)		78.3 (2.6)	11.6 (1.5)	89.9 (1.8)
	% in layer	0.0		93.8	71.1	89.9
Calb1 (*N* = 4)	% of all GFP^+^	16.0 (1.2)	2.2 (0.3)	38.7 (1.7)	7.3 (0.4)	64.2 (2.7)
	% in layer	82.2	31.0	73.3	35.4	64.2
Pdyn (*N* = 3)	% of all GFP^+^	11.9 (1.5)	2.8 (0.4)	29.5 (0.3)	3.6 (0.3)	47.7 (1.2)
	% in layer	80.8	13.6	51.9	44.2	47.7
All SOM (*N* = 17)	% of all SOM	11.5 (0.3)	2.0 (0.2)	30.9 (0.9)	9.5 (0.3)	54.0 (1.2)
	% in layer	80.3	17.9	61.3	40.0	54.0

In each subset, the top row indicates the number of FB-labeled GFP^+^ cells per layer as a percentage of all GFP^+^ cells counted in each brain, averaged by genotype. The SEM is indicated in parenthesis. The bottom row presents the same numbers expressed as a percentage of all GFP^+^ cells in each layer. Empty cells indicate that no GFP^+^ neurons were found in that layer. The bottom 2 rows of the table quantify the distribution of all retrogradely labeled SOM cells (both GFP^+^ and tdTomato^+^), averaged over all genotypes. These data are plotted in Figure 4*B*.

### Expression of protein markers in SOM subtypes

**Figure 5.**
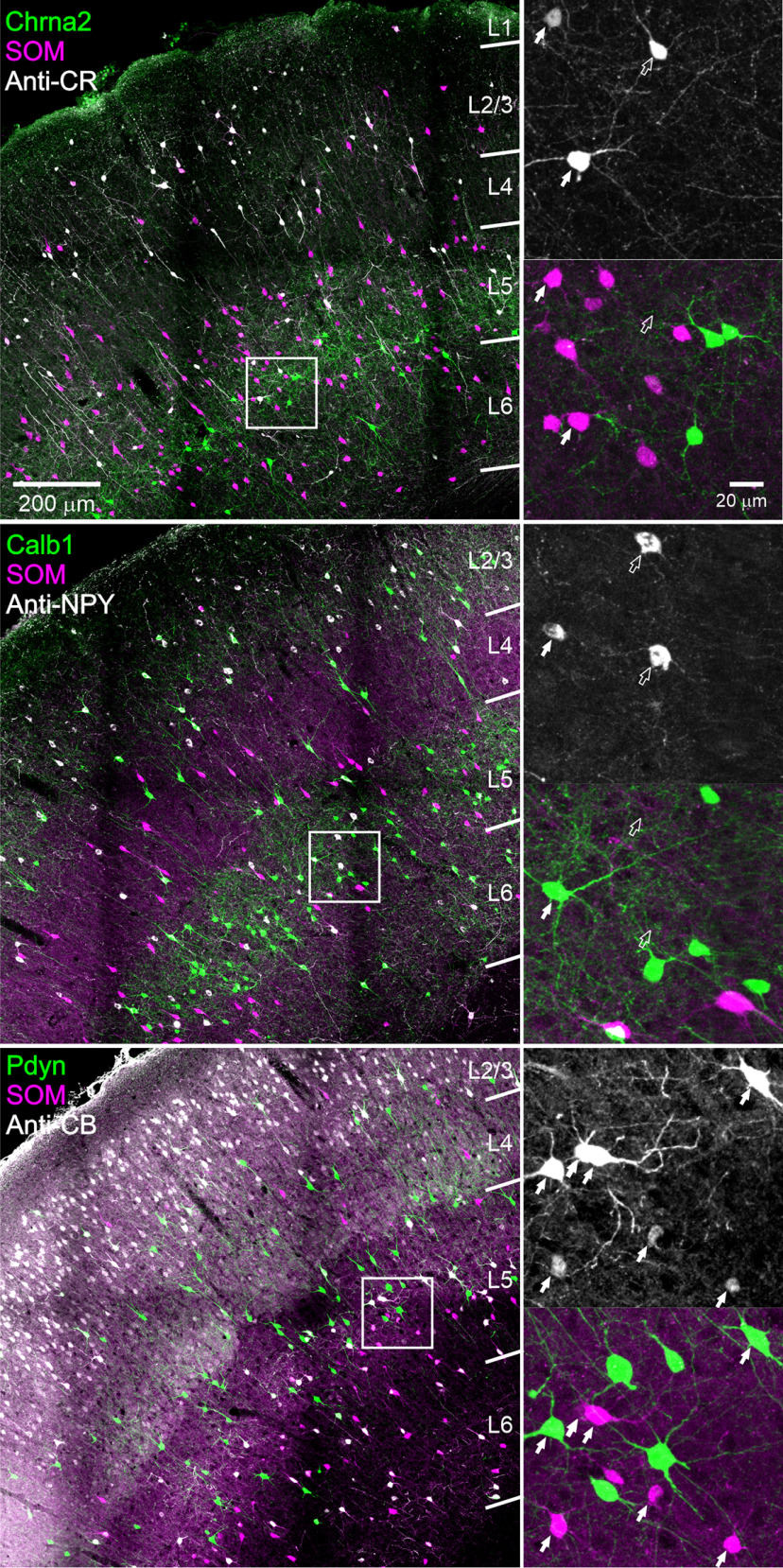
Immunostaining against 3 marker proteins. Top panels, Anti-CR immunostaining on a representative Chrna2 section. Middle panels, Anti-NPY immunostaining on a representative Calb1 section. Bottom panels, Anti-CB immunostaining on a representative Pdyn section. All images are projections of *z*-stacks taken with a 20×, 0.75 NA objective at 2.5 μ;m *z*-steps. Color channels were adjusted individually in each panel. For each section, one ROI is shown enlarged on the right, separated into two panels by color channels. Filled arrows indicate immunopositive SOM cells (GFP^+^ or tdTomato^+^), and hollow arrows indicate immunopositive non-SOM cells.

**Figure 6.**
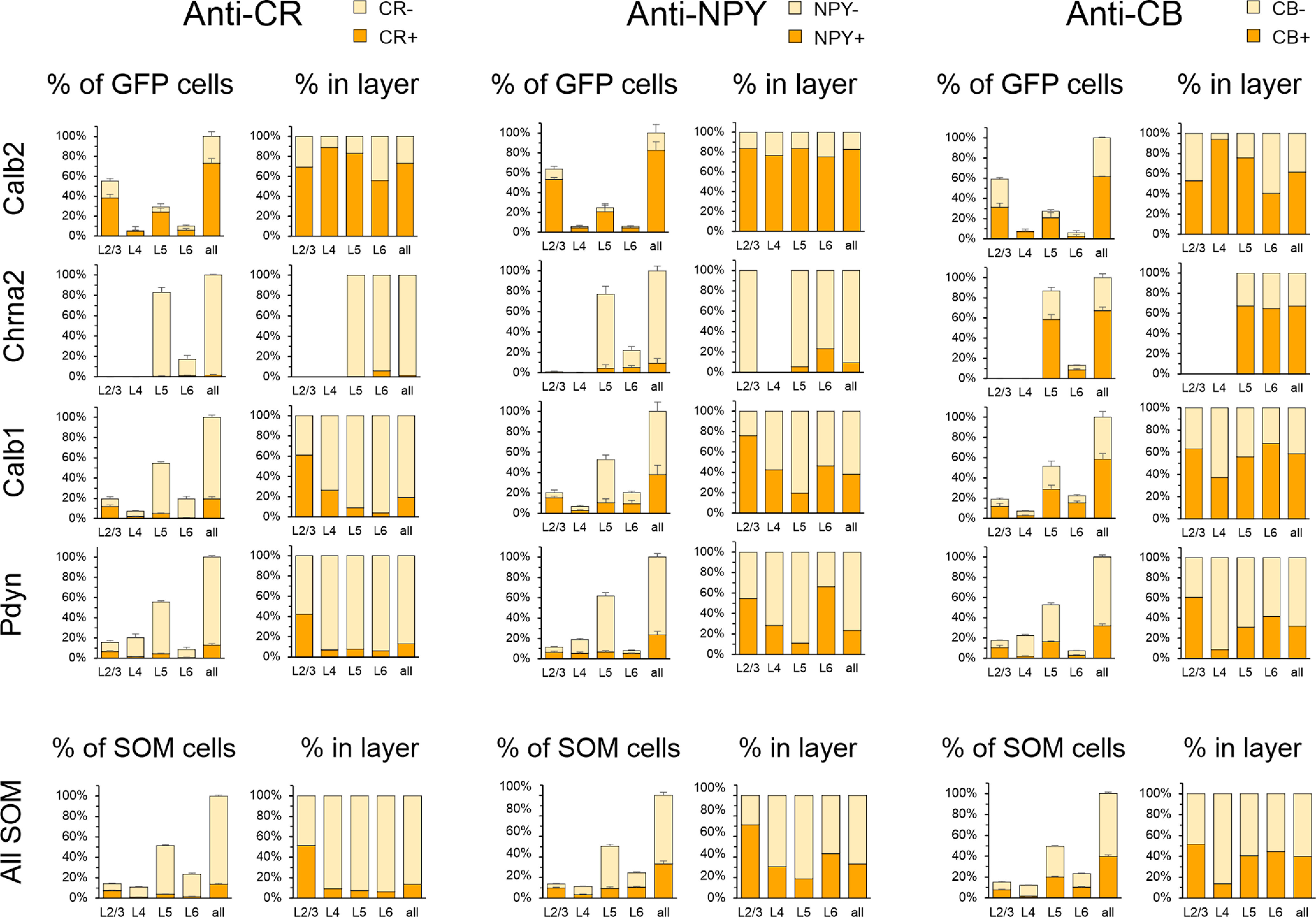
Protein marker expression in the 4 intersectional subsets. Left panels in each antibody block, Percentages of CR, NPY, and CB immunostained cells in each layer of all GFP^+^ cells counted in each brain, averaged within each genotype (top 4 rows), or of all GFP^+^ and tdTomato^+^ cells counted in each brain, averaged over all genotypes (bottom row). Error bars are the SEM. Right panels, Same numbers expressed as percentages of all GFP^+^ cells (top 4 rows) or of all GFP^+^ and tdTomato^+^ cells (bottom row) counted in each layer. *N* = 3 mice/genotype, 12 mice total. Numerical data are provided in Table 5.

Cortical interneurons can be differentiated by their pattern of expression of a variety of calcium-binding proteins and neuropeptides (Demeulemeester et al., 1988; Baimbridge et al., 1992; Kubota et al., 1994). We tested the four intersectional subsets for immunostaining against three proteins known to be expressed by SOM interneurons in the mouse: CR (product of the Calb2 gene), NPY, and CB (product of the Calb1 gene; Xu et al., 2010). Each antibody was tested on (typically) four sections/brain from three brains/genotype; only one antibody was tested on each section. Three representative sections stained with the three antibodies, respectively, are shown in Figure 5, with one L5 ROI in each section shown enlarged to the right of the low-power image. As for FB, we characterized each GFP or tdTomato-expressing cell as positive or negative for the antibody tested on that section by examining all optical planes taken through the section (Fig. 5, right panels). For each intersectional subset, we quantified the fraction of all GFP-expressing neurons labeled by each antibody in each layer, averaged over the three brains. These counts are plotted in Figure 6 (left panels) in each antibody block. The same data are also plotted in Figure 6 (right panels), normalized to the average count in each layer, for clarity. We also quantified the fraction of all SOM cells (both GFP and tdTomato expressing) immunostained by each antibody, averaged over all 12 brains. These data are plotted in the bottom row of Figure 6 in the same manner, with the right plot in each pair of plots showing the averaged counts normalized by layer. The full numerical dataset is provided in Table 5.

**Table 5:** Marker protein immunoreactivity in the 4 intersectional subsets

		L2/3	L4	L5	L6	All layers
Calb2 (*N* = 3)						
CR^+^	% of all GFP^+^	38.2 (3.7)	4.8 (4.8)	24.3 (3.7)	5.7 (1.8)	73.1 (4.6)
	% in layer	69.3	88.9	83.2	55.9	73.1
NPY^+^	% of all GFP^+^	53.1 (2.0)	4.4 (1.5)	20.6 (6.5)	4.4 (1.5)	82.5 (8.6)
	% in layer	83.4	76.5	83.5	75.0	82.5
CB^+^	% of all GFP^+^	31.3 (4.0)	7.1 (2.6)	20.7 (5.3)	2.4 (1.8)	61.5 (0.5)
	% in layer	53.0	93.9	75.8	40.4	61.5
Chrna2 (*N* = 3)						
CR^+^	% of all GFP^+^			0.4 (0.4)	1.0 (0.6)	1.4 (0.7)
	% in layer			0.5	6.0	1.4
NPY^+^	% of all GFP^+^	0.0 (0.0)		4.3 (3.7)	5.1 (2.0)	9.4 (4.7)
	% in layer	0.0		5.6	23.3	9.4
CB^+^	% of all GFP^+^			58.6 (4.7)	8.5 (1.2)	67.1 (3.7)
	% in layer			67.4	64.9	67.1
Calb1 (*N* = 3)						
CR^+^	% of all GFP^+^	11.7 (1.7)	1.9 (0.3)	4.8 (0.6)	0.8 (0.3)	19.2 (2.3)
	% in layer	61.0	26.4	8.9	4.0	19.2
NPY^+^	% of all GFP^+^	15.3 (1.6)	2.9 (0.7)	10.4 (3.8)	9.4 (3.3)	38.0 (9.2)
	% in layer	76.2	42.5	19.7	46.3	38.0
CB^+^	% of all GFP^+^	11.9 (2.8)	2.7 (0.6)	28.7 (4.2)	15.2 (1.8)	58.6 (5.6)
	% in layer	62.9	37.4	55.9	67.9	58.6
Pdyn (*N* = 3)						
CR^+^	% of all GFP^+^	6.6 (0.9)	1.4 (0.3)	4.4 (0.5)	0.5 (0.3)	13.0 (1.5)
	% in layer	42.3	7.0	8.0	6.1	13.0
NPY^+^	% of all GFP^+^	6.3 (1.3)	5.3 (1.3)	6.7 (1.4)	5.2 (0.5)	23.5 (3.6)
	% in layer	54.6	28.1	10.9	66.3	23.5
CB^+^	% of all GFP^+^	10.6 (2.3)	2.0 (0.4)	16.4 (0.7)	3.1 (0.4)	32.0 (2.0)
	% in layer	60.6	8.7	31.1	41.6	32.0
All SOM						
CR^+^ (*N* = 12)	% of all SOM	7.3 (0.6)	1.0 (0.2)	3.8 (0.3)	1.5 (0.1)	13.6 (0.9)
	% in layer	51.5	9.0	7.4	6.4	13.6
NPY^+^ (*N* = 12)	% of all SOM	9.7 (1.0)	3.4 (0.6)	9.4 (1.4)	10.7 (0.9)	33.3 (3.0)
	% in layer	71.4	30.6	18.7	43.3	33.3
CB^+^ (*N* = 12)	% of all SOM	7.9 (0.6)	1.7 (0.2)	20.0 (0.9)	10.3 (0.6)	39.9 (1.4)
	% in layer	51.6	13.8	40.5	44.5	39.9

For each antibody, the top row indicates the count of immunostained cells in each layer, expressed as a percentage of all GFP^+^ cells in the same sections. The SEM is indicated in parenthesis. The bottom row expresses the same counts as a percentage of all GFP^+^ cells in the same layer. Empty cells indicate that no GFP^+^ neurons were found. *N* = 3 mice for each antibody in each genotype; most brains were used for 2–3 antibodies each. The bottom block in the table indicates counts of immunostained cells expressed as a percentage of all SOM cells (GFP^+^ and tdTomato^+^) counted in each brain, averaged over all genotypes, *N* = 12 mice/antibody. These data are plotted in Figure 6.

In the Calb2 subset, ∼75% of neurons were CR^+^, with some minor variations between layers, as reported in a previous study of this intersection (Nigro et al., 2018). That not all Calb2 cells expressed CR, although Cre and CR proteins were presumably translated from a single bicistronic transcript, could mean that some neurons that expressed CR and Cre during prenatal or early postnatal development underwent Cre-mediated recombination but no longer expressed the marker protein in the adult animals tested here. The majority (∼60%) of Calb2 cells were also immunopositive for CB, a fraction very similar to that of triple-labeled CB^+^, CR^+^, and SOM^+^ cells of all CR^+^ and SOM^+^ cells (Nigro et al., 2018). An even larger fraction of Calb2 cells (∼80%) were NPY^+^, suggesting that most Calb2 cells expressed both CR and NPY. High coexpression of CR and NPY in upper layers SOM cells was previously noted in the cingulate cortex (Riedemann et al., 2016).

In contrast to Calb2 cells, two-thirds of Chrna2 SOM cells were immunopositive to CB but only 10% to NPY and nearly none to CR, indicating that the Calb2 and Chrna2 subsets were distinct populations with little or no overlap. In the Calb1 subset, ∼20%, 40%, and 60% of cells were immunopositive for CR, NPY, and CB, respectively. Lastly, Pdyn SOM cells were immunopositive to the three markers at about half the rates of Calb1 cells (13%, 23% and 32%, respectively). Especially low levels (<10%) of CR and CB immunoreactivity were found in L4 Pdyn cells, consistent with the absence of these markers from L4 X94 neurons (Ma et al., 2006; Naka et al., 2019).

In all SOM cells (Fig. 6, bottom row), the highest incidence of the three markers was found in L2/3, where at least 50% of SOM cells were immunopositive for each marker (tested separately). Fractions were lower in the other layers, especially for CR, which was detected in <10% of SOM cells in each of the other layers. A very similar laminar distribution of CR^+^ SOM cells in mouse S1 was reported previously by Xu et al. (2010). Notably, however, our counts for NPY in SOM cells (70% immunopositive in L2/3, 33% overall) were several folds higher than reported by Xu et al. (2010; ∼10% and 7%, respectively). This discrepancy could reflect the low sensitivity of the anti-NPY antibody used by Xu et al. (2010). Indeed, in a previous study by the same authors using the same antibody (Xu et al., 2006), no NPY expression was detected in GIN cells, a subset of GFP-expressing SOM interneurons in L2/3 and L5, whereas two other studies (in S1 and in cingulate cortex), using two NPY antibodies different from the ones used by Xu et al. (2006, 2010) or in the current study, found that ∼30% of L2/3 GIN cells were NPY^+^ (Ma et al., 2006; Riedemann et al., 2016). That NPY expression was strongly genotype dependent (e.g., >80% of all Calb2 cells, but <10% of Chrna2 cells were immunopositive for NPY) also indicated that our anti-NPY staining was both sensitive and specific. Lastly, a recent study using the same NPY antibody used here found that ∼50% of all SOM cells in adult mouse S1 cortex were NPY^+^ (Asgarian et al., 2022). We conclude that the NPY antibody we used provided a reliable estimate of NPY expression in SOM interneuron subsets.

### Multidimensional analysis of SOM subtypes

**Figure 7.**
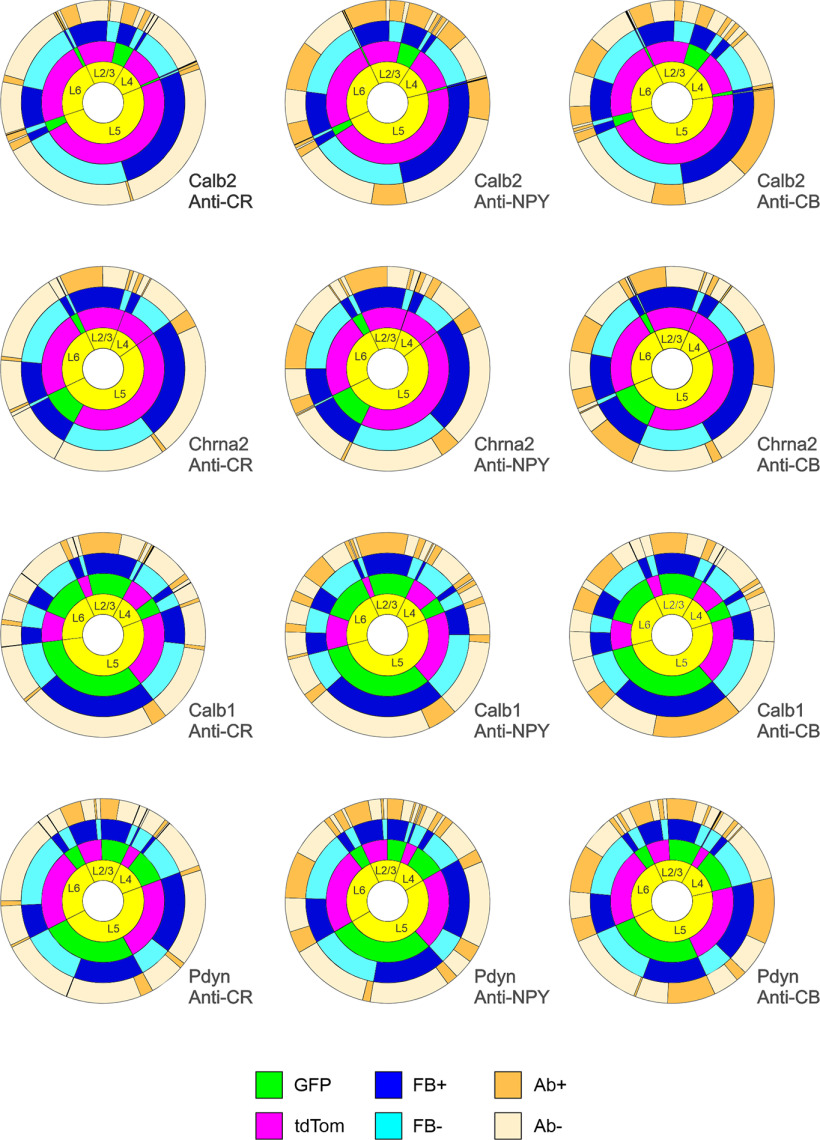
Sunburst charts illustrating the correspondence between fluorescent protein expression, retrograde labeling, and protein marker immunostaining. Ab, antibody. Each chart corresponds to one genotype stained with one antibody: *N* = 3 brains/chart. See text for a detailed explanation of the sunburst chart format. The numerical data used to generate these charts are provided in Extended Data Figure 7-1. An interactive version can be accessed at https://sites.google.com/view/somatostatinsubtypes/home.

The analysis above illustrates separately the fraction of neurons in each genetic subset labeled retrogradely from the pial surface (Fig. 4*B*) or immunostained against each of the protein markers (Fig. 6). However, since the same ROIs and the same neurons were used for both analyses, we could tag each neuron with the following four attributes: its laminar position, its genetic identity, its retrograde label and its protein marker expression, thus allowing us to examine correlations between these attributes. To convey these multidimensional data graphically, we present them in Figure 7 as a 4 × 3 grid of “sunburst charts,” with rows corresponding to the four genetic subsets and columns corresponding to the three protein markers. Each sunburst chart consists of four concentric rings representing the four attributes; the complete ring represents all SOM cells, and the angle subtended by each color sector is proportional to the fraction of cells expressing that attribute, averaged over three brains. Beginning in the inner ring with four sectors corresponding to four laminar positions, each sector is further split two ways with each consecutive ring: by fluorescent protein, by retrograde label, and by immunostaining, ending with 32 sectors in the outer ring corresponding to the 32 distinct combinations of the four attributes. The full numerical dataset is provided as Extended Data Figure 7-1. An interactive version of these plots can be accessed at https://sites.google.com/view/somatostatinsubtypes/home.

10.1523/ENEURO.0204-23.2023.f7-1 Figure 7-1 Summary data used to create the sunburst charts in Figure 6. The table indicates the average number of cells counted in each layer for each antibody, for every combination of genetic label (GFP^+^ or tdTomato^+^), retrograde label (FB^+^ or FB^–^), and immunolabel (Ab^+^ and Ab^–^), expressed as a fraction of all SOM cells (tdTomato expressing and GFP expressing) counted in the same sections. *N* = 3 for each antibody. Download Figure 7-1, XLS file.

As illustrated in Figure 7, Calb2 cells (green sectors in top row) were located mostly in L2/3, with a smaller population in L5. In both layers, most Calb2 cells were L1-projecting (dark blue sectors) and expressed CR, NPY, and CB (dark tan sectors); however, many non-Calb2 SOM cells were also CR^+^, and these were likely VIP-containing SOM interneurons (Gonchar et al., 2007; Xu et al., 2010). Chrna2 cells were located exclusively in L5/6, and nearly all were L1-projecting. They expressed CB but, unlike Calb2 cells, almost never expressed NPY or CR; therefore, these two subsets are nonoverlapping SOM populations. Calb1 cells comprised the majority of SOM cells in L2/3, L5, and L6. Nearly all Calb1 cells in L2/3, and the majority in L5, were L1-projecting. Most L2/3 cells in this subset were positive for all three markers, while in L5 about half expressed CB but only a small minority expressed CR or NPY. Note that while only 60% of Calb1 cells were CB^+^, virtually all CB^+^ SOM cells belonged to the Calb1 group, implying that the Calb1 group is a superset that includes Calb2, Chrna2 and likely other SOM subsets. Pdyn cells were located in all layers, comprising at least half of all SOM cells in L2/3, L4, and L5. Most of L2/3 Pdyn cells were L1-projecting, as indeed were most other SOM cells in this layer; however, only about half of L5 Pdyn cells, and nearly none in L4, were L1-projecting, consistent with the overlap of this subset with L4 targeting (non-Martinotti) X94 cells in these layers. The majority of Pdyn SOM cells in L2/3 were positive for each of the three protein markers, but nearly none of those in L4 or in the FB^–^ sector in L5 were immunopositive, consistent with the known absence of any of the marker proteins in X94 cells (Ma et al., 2006; Naka et al., 2019). We conclude from our multidimensional data that the Calb2 and Chrna2 subsets are relatively small, homogeneous, and disjoint subsets, while the Calb1 and Pdyn subsets are larger (each comprising close to, or about half, of all SOM interneurons) and nonhomogeneous. The Calb1 is likely a superset that includes the Calb2 and Chrna2 subsets, and the Pdyn group is likely a superset that includes the non-Martinotti X94 subtype.

### Electrophysiological classification of SOM subtypes

SOM interneurons have been shown to exhibit a diversity of electrophysiological properties, allowing (in some studies) their parcellation into subtypes that also exhibit distinct morphologic and neurochemical phenotypes, or distinct gene expression patterns (Halabisky et al., 2006; Ma et al., 2006; McGarry et al., 2010; Riedemann et al., 2018; Gouwens et al., 2020). Given the clear separation between the X94, Chrna2, and Calb2 subsets based on neurochemical markers, it was therefore of interest to examine their electrophysiological phenotypes, and to test how well they can be classified based on these properties. Also, given the high prevalence of non-Martinotti L4 and L5 cells in the Pdyn subset, it was important to compare electrophysiological characteristics of Pdyn neurons to those of the previously characterized X94 subtype (Ma et al., 2006). We therefore recorded *ex vivo* from Calb2 (*N* = 15 cells in 7 animals), Chrna2 (*N* = 36 cells in 22 animals), Pdyn (*N* = 23 cells from 6 animals), and X94 (*N* = 17 cells from 8 animals) neurons, focusing on L5, where all four subsets overlapped. For recording from Calb2 neurons, we used the same triple-transgenic genotype used for the histologic analysis, but for recording from the three other SOM subsets we also used crosses between the respective Cre driver, the Ai9 reporter, and the X94 (or in a few cases X98) lines, in which we recorded from both tdTomato-expressing Chrna2 or Pdyn cells and GFP-expressing X94 neurons in the same slices, often pairwise. We did not include the Calb1 intersection in these experiments because our analysis above indicated that this subset likely included the Calb2 and Chrna2 subsets, and possibly other L1-projecting SOM neurons in L5.

**Figure 8.**
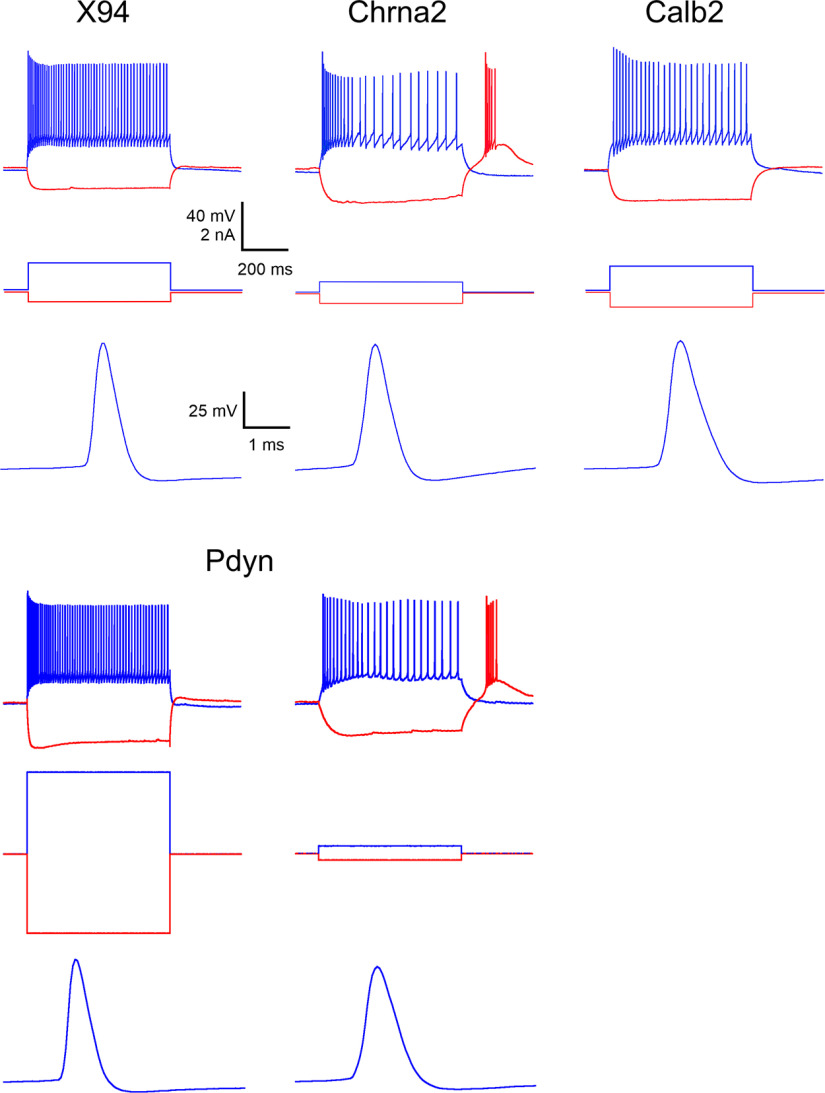
Characteristic firing patterns of the 3 intersectional subsets and of X94 cells. For each genotype, the top panel illustrates the response to a suprathreshold current step (blue trace) superimposed on a hyperpolarizing response to a negative current step (red trace); the middle panel shows the applied current steps; and the bottom panel shows the waveform of a single action potential evoked at rheobase, at an expanded time scale. Note the narrow spike and high steady-state firing frequency of the X94 neuron; the rebound burst and high input resistance of the Chrna2 cell; and the low firing frequency and wider spike of the Calb2 cell. The Pdyn subset displayed a mix of the X94 and Chrna2 firing patterns.

As illustrated in Figure 8 (top panels), X94, Chrna2, and Calb2 SOM cells had distinct firing patterns. While all subsets exhibited pronounced spike rate adaptation during a 600 ms suprathreshold current step, each had unique features that distinguished it from the other two. X94 cells, previously characterized as “quasi-fast spiking” (Ma et al., 2006), had considerably lower input resistance, faster (narrower) spikes, and higher steady-state firing frequency compared with the other two subsets. Chrna2 neurons fired a characteristic low-threshold burst at the onset of the current step, and, as previously reported (Hilscher et al., 2017), most Chrna2 cells also fired a rebound burst on recovery from hyperpolarization; 72% of all Chrna2 cells fired a rebound burst of two or more spikes, but only one (7%) of the Calb2 cells and none of the X94 cells did. Chrna2 cells also had the highest values of input resistance [Fig. 8 (note the low-amplitude current steps applied to Chrna2 cells)]. In both of these properties, Chrna2 cells resembled the previously characterized X98 subset (Ma et al., 2006). Calb2 cells fired at considerably lower rates compared with the other two subsets, a difference that was especially pronounced at the beginning of the current step, before spike frequency adaptation took place.

Unlike the relatively homogeneous properties of these three subsets, Pdyn neurons exhibited a dichotomy: some had X94-like firing patterns and spike waveforms, and the rest resembled Chrna2 neurons (Fig. 8, bottom panels), suggesting that this group included neurons from both the X94 and the Chrna2 subsets. While our histologic analysis suggested that the Pdyn group is likely to also contain Calb2 cells (based on NPY and CR immunostaining), we did not encounter Pdyn cells with Calb2-like electrophysiological phenotype, possibly because Calb2 cells are a small minority (5%) of L5 SOM cells (Fig. 1*B*).

**Figure 9.**
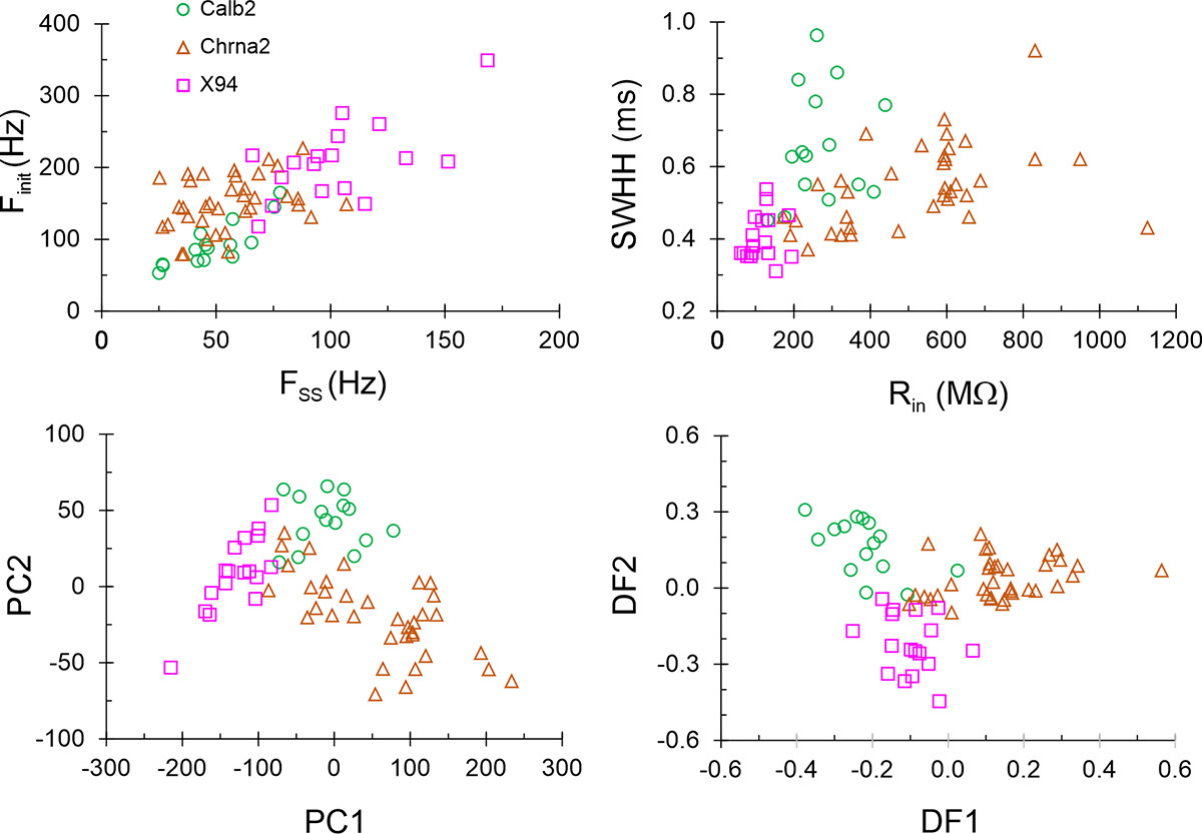
Multivariate analysis of electrophysiological properties in 3 genetically defined SOM subtypes. Top left, Plot of initial firing frequency versus steady-state frequency; top right, spike width at half-height versus input resistance; bottom left, the second principal component versus the first; bottom right, the second discriminant function versus the first. The full dataset of electrophysiological parameters is provided in Extended Data Figure 9-1.

To arrive at an objective classification of L5 SOM cells by their electrophysiological phenotype, we quantified 10 intrinsic electrophysiological parameters for each Calb2, Chrna2 and X94 cell, in addition to rebound spiking (see Materials and Methods). Other than spike height and sag, all parameters were significantly different in a three-way comparison (permutation test on the *F*-statistic), with four of them (*R*_in_, *SWHH*, *F*_init_, and *F*_SS_) different at the *p* < 0.0001 level, and *AR* different at the *p* < 0.0005 level. Plots of *F*_init_ versus *F*_SS_, and *SWHH* versus *R*_in_ (Fig. 9, top panels) largely separated the three subsets, but with some overlap between them. We then applied to the full dataset of 10 parameters two different dimensionality reduction methods: principal component analysis (PCA) and discriminant function analysis (DFA; Manly, 2005; Ma et al., 2006; Druckmann et al., 2013; Fig. 9, bottom panels). PCA is agnostic to the categorical identity (genotype) of each cell, while DFA is designed to maximize the separation in parameter space between precategorized groups. Both methods resulted in good separation of the datapoints but left a small central “zone of confusion” where datapoints of all three subtypes overlapped. Interestingly, although rebound bursting was not included as a parameter in this multivariate analysis, six of the Chrna2 cells that did not fire a rebound burst were within this zone of confusion. Including the Pdyn subset in this analysis resulted in Pdyn datapoints scattered within both Chrna2 and X94 point clouds (data not shown). The full dataset of electrophysiological parameters is provided as Extended Data Figure 9-1.

10.1523/ENEURO.0204-23.2023.f9-1 Figure 9-1 Summary of electrophysiological parameters measured in 91 neurons from four genetic subsets. See Materials and Methods for parameter definitions. In addition to the 10 parameters used in the multivariate analysis, the table also lists the number of rebound spikes fired upon recovery from a hyperpolarizing current step; the results of applying the Decision Tree classifier in Figure 9, with wrong predictions highlighted in red; and the values of the largest two principal components and the largest two discriminant functions for each cell. At the top of the table are the *p*-values calculated for each parameter in a 3-way comparison of the three subtypes and in three separate 2-way comparisons. For 10 Pdyn cells, *R*_in_ could not be calculated due to technical issues with the current monitor during the experiment, and these cells are therefore not included in the multivariate analysis. Download Figure 9-1, XLS file.

**Figure 10.**
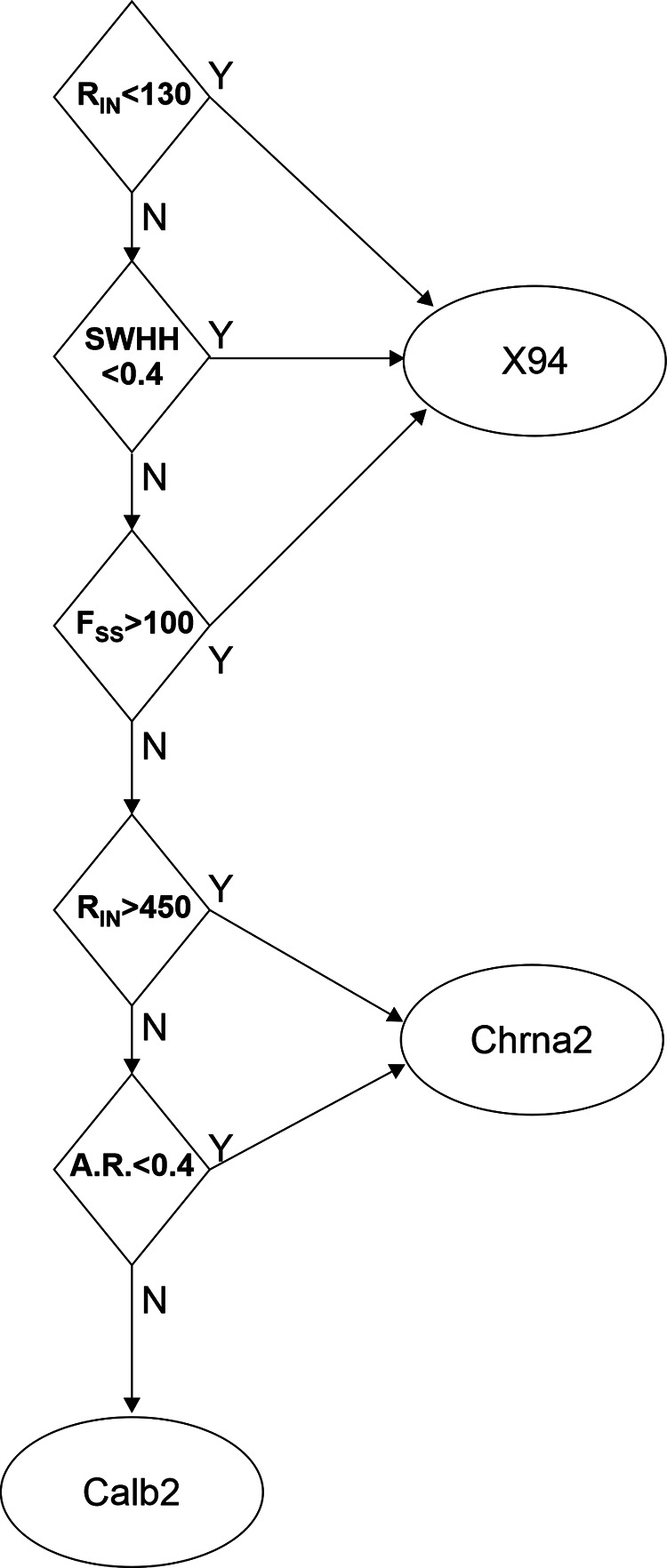
A decision tree for classifying the 3 L5 SOM subtypes by four basic electrophysiological properties. See Materials and Methods, subsection Parameter definitions. Units are *R*_in_, MΩ; SWHH, ms; *F*_ss_, Hz; AR, dimensionless.

Previous machine-learning approaches found the classification of cell types by electrophysiological properties alone to be challenging. For example, using a set of 44 electrophysiologically derived parameters, a random forest classifier achieved only 60% accuracy in assigning neurons to their correct transcriptomic type (Gouwens et al., 2020), and a K-nearest-neighbor classifier was unable to correctly separate Chrna2 from Calb2 neurons (Wu et al., 2022). To test how well electrophysiological properties can predict cell subtype, we constructed a simple decision tree with five nodes (decision points), using as input four of the parameters different at the *p* < 0.0005 level (Fig. 10). The first three nodes classified correctly all 17 X94 cells but misclassified two of the 36 Chrna2 cells as X94. The last two nodes correctly classified all 15 Calb2 cells and all but three of the remaining Chrna2 cells (which were misclassified as Calb2). Again, although rebound bursting was not used as a decision parameter, all five misclassified Chrna2 cells did not fire a rebound burst. In all, the decision tree classified correctly 86% of Chrna2 cells and 93% of the full dataset; these classification results are included in Extended Data Figure 9-1. We conclude that the three genetically defined SOM subtypes we characterized here can be classified with >90% accuracy using a small number of basic electrophysiological properties.

## Discussion

In an earlier study (Ma et al., 2006) we identified two SOM subtypes, labeled by GFP expression in the X94 and X98 transgenic lines. X98 cells were L1-targeting, had high input resistance and relatively slow spikes, fired low-threshold spike bursts, and expressed CB and (variably) NPY. X94 cells were L4-targeting, had lower input resistance and faster spikes, fired at high frequencies, and did not express these protein markers. More recently, Hilscher et al. (2017) described Chrna2 neurons in L5 as L1-targeting cells that fired low-threshold bursts; in this they resembled X98 cells, but no marker proteins (other than somatostatin) were tested. An *in vivo* recording study (Muñoz et al., 2017) identified two morphologic types of SOM cells in L5, in addition to L4-projecting non-Martinotti SOM cells: “T-shaped,” with a single main axon extending to L1 before branching, and “fanning-out,” with multiple ascending axon collaterals and with dense arborizations in both L2/3 and L1. These three morphologic types also exhibited distinct behaviorally linked activity patterns. A subsequent *ex vivo* study (Nigro et al., 2018) observed the same three morphologic types and found fanning-out cells preferentially among the Calb2 subset.

The studies above were conducted by three different laboratories using disparate methods and left several questions unanswered. Are the Chrna2, Calb2, and Calb1 subsets disjoint or overlapping? What protein markers are differentially expressed between them? What fraction of the L5 SOM population is captured by each of them? How are these intersectionally defined subsets related to the previously characterized X94 and X98 subsets? Lastly, how do all of these SOM subsets map onto the recent multimodal MET taxonomy, developed by yet another group of investigators? Here we set out to address these questions by examining all five previously studied genotypes and one novel genotype side by side. We found a clear separation between three SOM subsets—Chrna2, Calb2, and X94—which constitute nonoverlapping populations and differ from each other in both categorical and quantitative properties. As illustrated in Figures 1-6 and summarized graphically in Figure 7, the Chrna2 subset resides in L5B, while Calb2 neurons are split 2:1 between L2/3 and L5/6. Both subsets are L1-projecting (Martinotti) neurons and express CB, but, unlike Chrna2 cells, most Calb2 cells also express CR and NPY, indicating that these two subsets are fully disjoint. These characteristics also set them apart from X94 cells, which reside in L4/5, are L4-projecting, and do not express any of these protein markers (Ma et al., 2006; Naka et al., 2019). These three genetically defined subsets also have distinct firing patterns (Fig. 8) and segregate into largely nonoverlapping clusters by their electrophysiological parameters (Figs. 9, 10).

Notably, this segregation is not perfect: about one-quarter of all Chrna2 cells did not exhibit the strong rebound burst, which characterized the remaining cells in this group, and the same nonbursting cells were found by multivariate analysis of electrophysiological parameters (which did not include rebound bursting as input) to overlap with cells from the other two subtypes. Also, a “decision tree” classifier, based on electrophysiological parameters that did not include rebound bursting, assigned 14% of Chrna2 cells—all nonbursting—into one of the two other groups. This could be interpreted as “leakiness” in the expression of the Cre allele (i.e., some cells expressing Cre even when the respective promoter is not activated), but could also reflect a true biological continuum or gradient in the expression of electrophysiological properties, as opposed to strict separation into taxonomic entities with distinct phenotypes (Cembrowski and Menon, 2018). A genetic and epigenetic basis for such a continuum was recently suggested by large-scale transcriptomic studies (Tasic et al., 2016; Gouwens et al., 2020; Scala et al., 2021; Yao et al., 2021b).

The caveats above notwithstanding, the multimodal correspondence of axonal target, neurochemical markers, and electrophysiological properties (summarized in the Visual Abstract and in Table 6) strongly support consideration of the genetically defined Calb2, Chrna2, and X94 subsets as *bona fide* neuronal subtypes of the SOM subclass. In contrast, the genetically defined Calb1 and Pdyn subsets are nonhomogeneous, and seem to comprise a mix of SOM neurons from the other three subtypes.

**Table 6:** Comparison of the main characteristics of the three L5 SOM subtypes

	Laminar position	Axonal target	Protein markers	Electrophysiological characteristic	SWHH IQR (ms)	R_in_ IQR (MΩ)	F_init_ IQR (Hz)	F_SS_ IQR (Hz)
X94	L4, L5	L4		Quasi fast-spiking	0.35–0.45	90–130	170–220	85–115
Chrna2	L5/6	L1	CB	High R_in_, rebound bursts	0.45–0.6	340–610	130–180	40–70
Calb2	L2/3, L5	L1, L2	CB, CR, NPY	Low F_init_, wider spikes	0.5–0.8	220–300	70–100	40–60

Approximate interquartile range (IQR; 25th to 75th percentiles) is listed for each of the 4 basic parameters plotted in Figure 9. IQRs were largely nonoverlapping between subsets, except for partial overlap of SWHH and the complete overlap of *F*_SS_ between Chrna2 and Calb2.

**Table 7:** Suggested correspondence of SOM subtypes with the Sst-MET taxonomy (Gouwens et al., 2020)

MET 1	MET 2	MET 3	MET 4
Long-range	(L2/3 Calb2)	L2/3 Calb2	L5 Calb2
MET 5	MET 6	MET 7	MET 8
(X98)	Chrna2	L5 X94	L4 X94

Intersectional and transgenic subtypes are listed under the most comparable Sst-MET type; when listed in parenthesis, the correspondence is conjectural. Only Sst-MET types 1–8 are included.

It is informative to estimate how many of all SOM cells, and specifically of L5 SOM cells, belong to these three largely disjoint subtypes. The Calb2 and Chrna2 groups together account for 27% of L5 SOM cells and 23% of all SOM cells (Table 1). To estimate the size of the X94 subset, we assume that it consists of all FB^–^ Pdyn cells in L4 and L5, which comprise 26% of L5 SOM cells and 21% of all SOM cells (Extended Data Fig. 7-1). This assumption may be an underestimate, as some X94 cells may be retrogradely labeled by FB (14% of X94 cells in all layers; Fig. 3), but it may also be an overestimate, as some non-L1-targeting Pdyn cells in L5 may belong to other subtypes. Keeping these uncertainties in mind, we estimate that the three subtypes together account for >50% of L5 SOM cells, and for >40% of all SOM interneurons.

How do these three SOM subtypes fit within the recent multimodal MET classification (Gouwens et al., 2020)? A likely correspondence is presented in Table 7. The L2/3 and L5 members of the Calb2 subtype appear to correspond to Sst-MET types 3 and 4, respectively, based on CR expression, L1-targeting, and laminar position; some L2/3 Calb2 cells may also be included in Sst-MET 2. Chrna2 cells likely correspond to Sst-MET type 6, based on Chrna2 expression, L1-targeting, and laminar position. Interestingly, the Sst-MET 5 type expresses lower levels of Chrna2 but shares with Sst-MET 6 high expression of CoupTFII/Nr2f2 and contains L5 neurons with a pronounced bursting phenotype (Gouwens et al., 2020). We speculate that X98 cells, which have a bursting phenotype but are largely distinct from the Chrna2 subset (Fig. 2), may belong to the Sst-MET 5 type. L4 X94 cells seem to correspond to Sst-MET type 8, based on their laminar position, dense axonal arbor in L4, and *Hpse* expression (Naka et al., 2019), although in visual cortex (used for the MET classification) they also have substantial axonal arborization in L1 (Scala et al., 2019). L5B X94 cells likely correspond to Sst-MET 7, based on L4-targeting and *Hpse* expression; but there are also some *Hpse*-expressing, L4-targeting infragranular SOM cells in Sst-MET 11 and 12. Notably, the descriptors “T-shaped” and “fanning out” do not map onto unique MET types; for example, Sst-MET 4, 6, and 7 contain T-shaped cells, while most Sst-MET 4 and 5, and some Sst-MET 9 and 12 cells, are fanning-out cells (Gouwens et al., 2020). Thus, we suggest that these terms be used as morphologic descriptors and not as subtype designations.

Our study is the first to apply retrograde labeling from an epipial dye deposit to identify L1-projecting interneurons in the mouse, although a previous study has done so in the rat (Ramos-Moreno and Clascá, 2014). L1-projecting SOM interneurons with radially ascending axons are historically referred to as Martinotti cells (Marín-Padilla, 1990; DeFelipe, 2002), and some earlier studies regarded “Martinotti” and “somatostatin containing” as synonymous terms. That not all SOM interneurons are L1-projecting was made clear by the parallel discovery of two non-L1-projecting SOM groups: the long-range-projecting, sleep-active, NPY/nNOS-expressing cells in L2 and L6 (Tomioka et al., 2005; Gerashchenko et al., 2008); and L4-projecting X94 cells in L4 and L5B (Ma et al., 2006). The recent transcriptomic studies revealed additional SOM types that do not project to L1. Of the 13 Sst-MET types (Gouwens et al., 2020), four types (Sst-MET 10–13) have cell bodies in L5/6 and axonal arborization concentrated in L4–L6, with virtually no axonal projections in L1. This is in addition to Sst-MET types 1 and 8, which have only minor projections in L1. Thus, only about half of all Sst-MET types are *bona fide* Martinotti cells. The transcriptomic studies, however, are unable to estimate the relative abundance of different types, as cells in these studies were not sampled in an unbiased manner. Our retrograde labeling results provide, for the first time, an unbiased estimate of the fraction of mouse SOM interneurons with an axonal arbor in L1, which in our experiments was slightly >50% and varied by layer, from ∼80% in L2/3 to 60% in L5, 40% in L6, and <20% in L4. Interestingly, the previous rat study (Ramos-Moreno and Clascá, 2014) only identified 26% of SOM cells in S1 as retrogradely labeled after an epipial FB deposit, most of them in L2/3, with none in L6. Both studies used the same dye concentration, and survival time in the rat study was 7 d, compared with 24 h in the current study. It is possible that the thicker pia mater in the rat restricted the diffusion of dye (and thereby dye uptake) in this previous study.

In a study recently published in preprint form, Wu et al. (2022) used intersectional strategies to target various genetically defined SOM subsets, and examined intrinsic and synaptic electrophysiological properties of three of these subsets, including the Calb2 and Chrna2 subsets we studied here. Both studies are in good agreement on the overall fraction of Calb2 and Chrna2 of all SOM interneurons and on their laminar distributions. Our studies diverge, however, in respect to electrophysiological characteristics of these subsets. Wu et al. (2022) found no significant differences in input resistance and spike width between Calb2 and Chrna2 cells, whereas we found highly significant differences in these parameters (*p* < 0.0001 and *p* = 0.01, respectively, in pairwise comparisons), and also in the initial firing frequency and adaptation ratio (Extended Data Fig. 9-1). Consequently, our classifier could separate Calb2 and Chrna2 cells based on their electrophysiological properties with >90% accuracy (Fig. 10), whereas the classifier used by Wu et al. (2022) could not reliably separate these subsets. Some of these discrepancies may be attributable to differences in recording conditions (e.g., room temperature in Wu et al., 2022, vs 32°C in our study).

While potentially powerful, the strategy of using intersectional genetics to target specific SOM subtypes remains limited by available driver lines, as evident from the fact that most of the intersectional genotypes studied by Wu et al. (2022) labeled more than one transcriptomic “supertype” (Wu et al., 2022, their Table S3). Our study was subject to the same limitation; for example, the Calb2 intersection labels both L2/3 and L5 neurons, subsets that fall under the same transcriptomic supertype (Yao et al., 2021a) but under different MET types (Gouwens et al., 2020). While Wu et al. (2022) found that L2/3 and L5 Calb2 cells have similar electrophysiological properties, these subsets could still differ in their synaptic connectivity; indeed, it was suggested that L2/3 and L5 Martinotti cells preferentially target pyramidal cells with cell bodies in the same respective layer (Jiang et al., 2015). Thus, driver lines which target each of these MET types separately would be very useful. Importantly, neither the current study nor that by Wu et al. (2022) found a specific genetic strategy to target L4-projecting X94 cells (Ma et al., 2006); while the Pdyn subset is enriched in X94-like neurons, both studies found that it includes many L1-projecting cells as well (note, however, that the study by Wu et al., 2022, used a different Pdyn intersection than the one used here). Clearly, more specific driver lines and/or more sophisticated combinatorial reporters are needed to restrict reporter expression to smaller, more homogeneous populations. Once such genetic strategies are developed, they could be used to examine the local and long-range synaptic connections of the targeted populations, to document their pattern of activity in the behaving animal, and to test their involvement in sensory processing, motor planning, and cognition. While a daunting task, accomplishing it is essential if we are to establish which of the different transcriptomic groups proposed by recent taxonomies are *bona fide*, biologically meaningful neuronal subtypes, what roles each subtype plays in cortical computations, and how different subtypes contribute to animal and human behavior.

## Synthesis

Reviewing Editor: Arianna Maffei, Stony Brook University Decisions are customarily a result of the Reviewing Editor and the peer reviewers coming together and discussing their recommendations until a consensus is reached. When revisions are invited, a fact-based synthesis statement explaining their decision and outlining what is needed to prepare a revision will be listed below. The following reviewer(s) agreed to reveal their identity: NONE. Note: If this manuscript was transferred from JNeurosci and a decision was made to accept the manuscript without peer review, a brief statement to this effect will instead be what is listed below.

The authors provided a thorough response to the reviewers’ comments and edited the manuscript to provide additional detail and clarity.
